# Universal and Energy‐Efficient Approach to Synthesize Pt‐Rare Earth Metal Alloys for Proton Exchange Membrane Fuel Cell

**DOI:** 10.1002/advs.202305110

**Published:** 2023-11-20

**Authors:** Nannan Jiang, Bing Huang, Minghao Wang, Yumo Chen, Qiangmin Yu, Lunhui Guan

**Affiliations:** ^1^ CAS Key Laboratory of Design and Assembly of Functional Nanostructures & Fujian Key Laboratory of Nanomaterials Fujian Institute of Research on the Structure of Matter Chinese Academy of Sciences Fuzhou 350000 P.R. China; ^2^ Shenzhen Geim Graphene Center Tsinghua‐Berkeley Shenzhen Institute & Institute of Materials Research Tsinghua Shenzhen International Graduate School Tsinghua University Shenzhen 518055 P.R. China; ^3^ University of Chinese Academy of Sciences Beijing 100049 P.R. China

**Keywords:** oxygen reduction reaction, platinum‐rare earth metal alloys, proton exchange membrane fuel cell, rapid Joule thermal‐shock, stability

## Abstract

Traditional synthesis methods of platinum‐rare earth metal (Pt‐RE) alloys usually involve harsh conditions and high energy consumption because of the low standard reduction potentials and high oxophilicity of RE metals. In this work, a one‐step strategy is developed by rapid Joule thermal‐shock (RJTS) to synthesize Pt‐RE alloys within tens of seconds. The method can not only realize the regulation of alloy size, but also a universal method for the preparation of a family of Pt‐RE alloys (RE = Ce, La, Gd, Sm, Tb, Y). In addition, the energy consumption of the Pt‐RE alloy preparation is only 0.052 kW h, which is 2–3 orders of magnitude lower than other reported methods. This method allows individual Pt‐RE alloy to be embedded in the carbon substrate, endowing the alloy catalyst excellent durability for oxygen reduction reaction (ORR). The performance of alloy catalyst shows negligible decay after 20k accelerated durability testing (ADT) cycles. This strategy offers a new route to synthesize noble/non‐noble metal alloys with diversified applications besides ORR.

## Graphical abstract

In this study, a highly efficient one‐step strategy has been developed for the synthesis of Pt‐RE alloys using rapid Joule thermal‐shock (RJTS). Importantly, the method is universality for the preparation of a family of Pt‐RE alloys, including Ce, La, Gd, Sm, Tb, and Y. This method enables the production of Pt‐RE alloys within mere tens of seconds and at a low energy consumption of only 0.052 kW h, which is 2–3 orders of magnitude lower than the reported methods. Furthermore, the RJTS method allows for the precise regulation of alloy size within ≈5 nm, thereby delivering superior catalytic performance toward the oxygen reduction reaction and proton exchange membrane fuel cells.

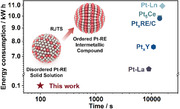

## 1 Introduction

The excessive reliance on fossil fuels not only wreaks havoc on the environment but also poses far‐reaching implications for human health.^[^ ^1^ ^]^ To combat these negative effects, it is imperative to develop clean energy such as hydrogen energy. Proton exchange membrane fuel cell (PEMFC) is a popular energy conversion device, which can efficiently convert hydrogen energy to electricity. The efficiency of energy conversion in PEMFC primarily relies on the catalytic performance of the ORR catalyst.^[^ ^2^, ^3^, ^4^ ^]^ Although Pt is widely used as an ORR catalyst in fuel cells, its high cost and tendency to aggregate limit its practical applications. Hence it is urgency to develop low‐cost and highly‐efficient catalysts with lower Pt loading.^[^ ^5^, ^6^, ^7^, ^8^, ^9^ ^]^

Modifying Pt‐based electrocatalysts through alloying is a promising approach to enhance their performance.^[^ ^10^, ^11^, ^12^, ^13^ ^]^ In the realm of this research domain, rare earth (RE) metals have garnered substantial interest due to their remarkable ability to regulate Pt catalytic activity.^[^ ^14^, ^15^, ^16^, ^17^, ^18^ ^]^ Pt‐RE alloy catalysts have demonstrated exceptional performance, surpassing that of most ORR catalysts.^[^ ^19^, ^20^, ^21^, ^22^ ^]^ Because alloying of Pt and RE enables them to lowered d‐band center of Pt, further optimizing the intermediate adsorption energy of Pt.^[^ ^23^ ^]^ However, the synthesis of high‐performance Pt‐RE alloy catalysts remains challenging primarily because of the large disparity in reduction potentials between Pt (Pt^2+^/Pt is 1.188 V) and RE (e.g., −2.336 V for Ce^3+^/Ce).^[^ ^22^ ^]^ The metallic RE exhibits an extraordinary affinity for oxygen owing to the strong formation of RE─O bonds. Therefore, synthesis typically requires conduction in an environment devoid of oxygen and moisture. The synthesis of Pt‐RE alloys reported thus far has been a tedious process involving preparing the precursor, using molten alkali as a reducing agent, and preventing O_2_ and H_2_O, together resulting in high energy consumption and poor controllability.^[^ ^24^, ^25^, ^26^, ^27^, ^28^ ^]^ Moreover, the long‐time synthesis of Pt‐RE alloy and the high surface energy of Pt make it prone to alloy aggregation, leading to uneven size distribution of the prepared alloys.^[^ ^4^ ^]^ For instance, Kanady et al. innovatively synthesized Pt_x_RE/C with average particle size larger than 20 nm at elevated temperatures.^[^ ^27^ ^]^ Hu et al. and Zhang et al. prepared Pt‐RE alloys on carbon carriers through chemical reduction, while this mild method also required high energy consumption and suffered from alloys aggregation issues.^[^ ^22^, ^29^ ^]^ Hence, it is challenging to prepare Pt‐RE alloys in a short time to avoid the alloys aggregated.

In this work, we develop a one‐step RJTS method for synthesizing Pt‐RE alloys, with an extraordinarily low energy consumption of 0.052 kW h and a short time within 100 s. The as‐synthesized Pt‐RE alloys are single alloy, uniform size (≈5.0 nm), as well as strong binding between alloy and substrate, ensuring their stability and durability. As a results, Pt‐RE alloys such as Pt‐Ce alloy displays excellent performance for ORR with a half‐wave potential (E_h_) of 0.91 V, high mass activity (MA) of 0.67 A mg_Pt_^−1^, which is 3.7 times higher than Pt/C. It demonstrates superior performance with a specific activity (SA) of 1.07 mA cm^−2^ at 0.9 V, ≈2.7 times higher than Pt/C. Furthermore, after self‐optimization process, the MA of Pt‐Ce increase by 9.1% and the SA increased by 5.7%, showing excellent stability. The density functional theory (DFT) calculations show the similar results with the experimental results. The Pt‐Ce alloy catalyst displays exceptional performance in an acidic H_2_/O_2_ PEMFC with a current density of 3.06 A cm^−2^ at 0.65 V and a peak power density of 2.17 W cm^−2^ which outperforms commercial Pt/C by two‐fold. Furthermore, after undergoing a meticulous stability test of 30 k cycles, it demonstrates outstanding durability with only a 21.7% decrease in MA from 0.69 A mg_Pt_^−1^ to 0.54 A mg_Pt_^−1^. This level of stability surpasses the DOE 2025 activity target of 0.44 A mg_Pt_^−1^.

## 2 Results and Discussion

### 2.1 Preparation and Characterization of Samples

**Figure 1:**
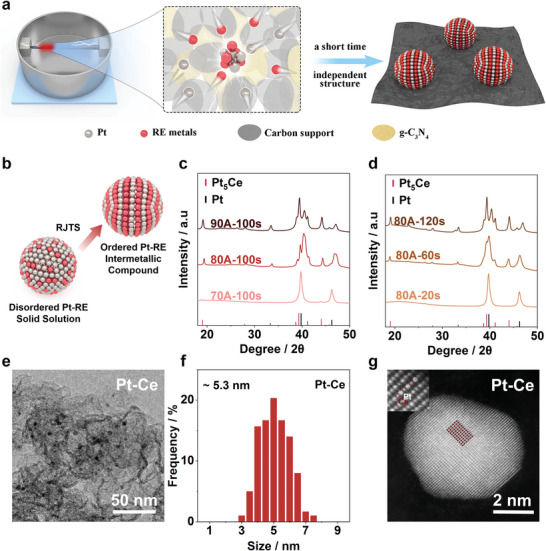
a) Schematic of the preparation process of Pt‐RE alloys. b) Model diagram of the transformation from solid solution to intermetallic compounds. c) The XRD patterns of Pt‐Ce alloy synthesized for 100 s at different currents. d) The XRD patterns of Pt‐Ce alloy synthesized at 80 A with different times. The reference patterns are Pt_5_Ce (JCPDS 65–8221), Pt (JCPDS 65–2868). e) The TEM image of Pt‐Ce alloy. f) The histogram of particle size distribution of Pt‐Ce alloy. g) The AC‐TEM image of Pt‐Ce alloy.

We develop a one‐step RJTS method to synthesize a family of Pt‐RE alloys (**Figure** **1**a) and this method providing many advantages in the synthesis process, such as direct solid‐phase mixing in air to prepare precursors, metal reduction under anhydrous conditions, and the synthesis of Pt‐RE alloy particles with embedded structures.^[^ ^30^, ^31^, ^32^ ^]^ Initially, RE metals dissolve in Pt clusters in a disordered state, and then transform to ordered intermetallic compounds by RJTS as shown in Figure 1b. The XRD patterns reveal that the intermetallic Pt‐Ce alloys appear when the current increases from 70 to 90 A (Figure 1c; Figure S1, Supporting Information), indicating that higher temperature increases thermal energy, thus enhancing the transformation of solid solutions into intermetallic compounds. According to the XRD spectra, Pt‐Ce is bulk polycrystalline, matching well with hexagonal Pt_5_Ce (PDF #65‐8221). The Pt_5_Ce with a hexagonal structure often exhibits superior ORR activity because it corresponds to the phase that exposing more Pt active sites.^[^ ^11^ ^]^ Figure 1d shows that even when the RJTS is conducted for varying time periods with a constant current of 80 A, the phase transition process remains clearly identifiable. This evidence confirms that the Pt and Ce exist in the form of metallic states during the synthesis process, further affirming the transformation from a solid solution to an intermetallic compound (Figures S2–S7, Supporting Information). Taking Pt‐Ce alloy as an example, Figure 1e illustrates that many Pt‐Ce alloys are individually embedded in carbon support, creating a robust alloy‐substrate structure. The average particle size of Pt‐Ce alloy is ≈5.3 nm (Figure 1f) with a narrow size distribution. Figure 1g is the spherical aberration corrected transmission electron microscope (AC‐TEM) image of Pt‐Ce alloy. The interplanar spacing of Pt‐Ce is 0.23 nm, corresponding to the (111) crystal facet of Pt_5_Ce. It reveals its regular atomic arrangement structure, further confirming that the Pt‐Ce alloy is intermetallic compounds. These results show that the high‐quality and highly‐ordered Pt‐Ce intermetallic compounds with uniform particle size and embedded structure can be synthesized in a very short period by RJTS.

**Figure 2:**
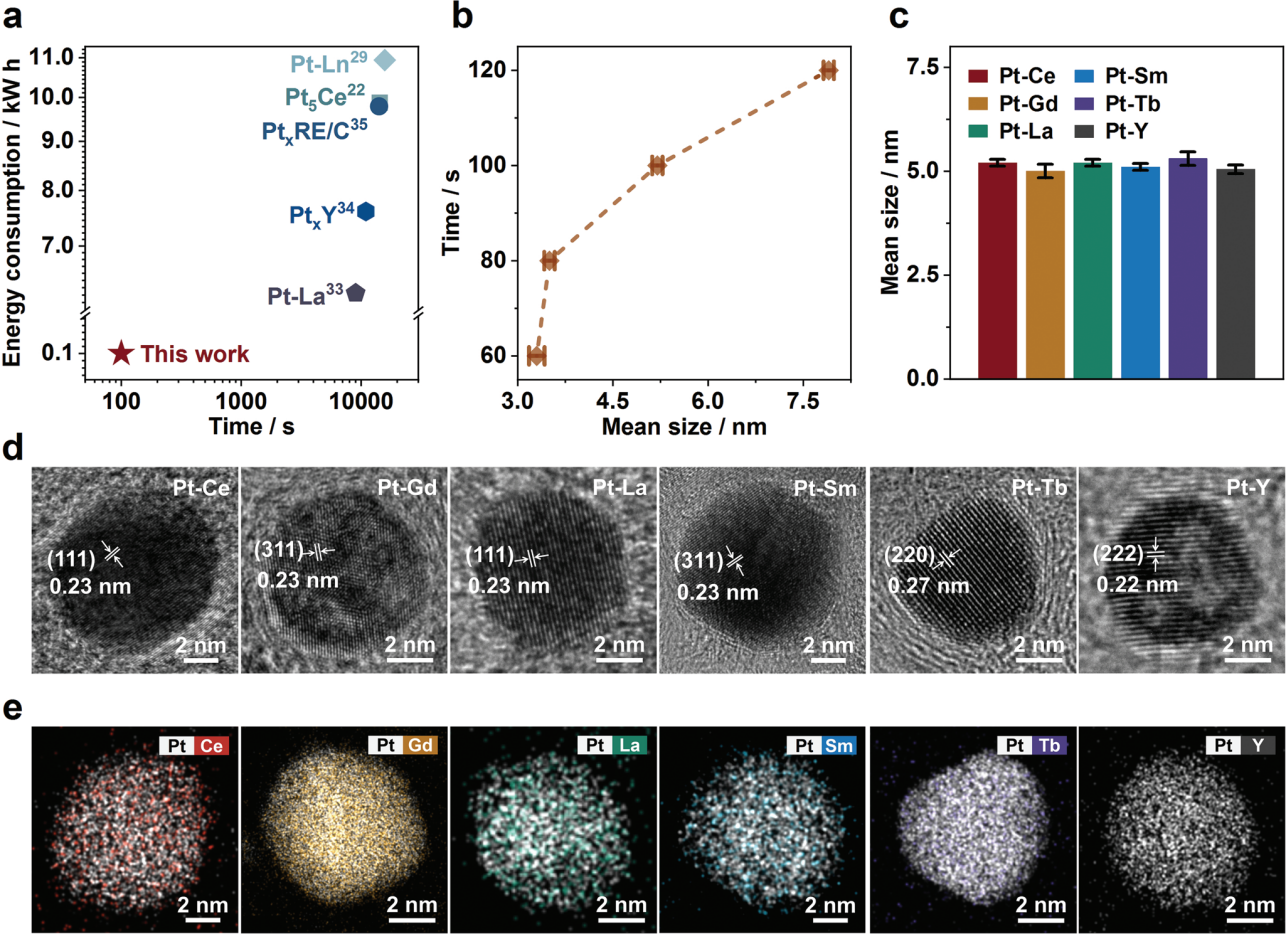
The advantages of RJTS method. a) Scatter plot comparing synthesis time, energy consumption with peers.^[^ ^22^, ^29^, ^34^, ^35^ ^]^ b) Point‐and‐line plot of particle size variation of Pt‐Ce alloy with reaction time. c) Histogram of average particle size for Pt‐RE alloy family. d) The HR‐TEM images of Pt‐RE alloy family synthesized at 80 A for 100 s. e) The elements mapping images of Pt‐RE alloy family synthesized at 80 A for 100 s.

The RJTS method exhibits significant advantages when compared with reported methods. First, the synthesis time of Pt‐Re alloys is only tens of seconds (**Figure** **2**a; Table S1, Supporting Information), which is 2–3 orders of magnitude shorter than that of other reported methods. Additionally, the energy consumption of this method is only 0.052 kW h, which is more than 200 times lower than that of some reported methods.^[^ ^22^, ^29^, ^33^, ^34^, ^35^ ^]^ Second, the particle sizes can be effectively controlled by thermal energy manipulation such as altering the reaction time or current. As seen in Figure 2b, under the specified conditions of 80 A, the particle size increases with prolonged processing time. The size distribution of Pt‐RE particles under different synthesis conditions is provided in Figures S8–S13 (Supporting Information), presenting matching crystal structures (presented with sample names), as well as uniform and embedded metal particles in the carbon support across all cases. Figure 2c shows that the average particle size of Pt‐RE alloys can be perfectly controlled at 5.0±0.5 nm. Figure 2d displays the HR‐TEM images of Pt‐RE alloys family with clear lattice fringes, indicating a successful synthesizing process of a series of high‐quality Pt‐RE alloys. For instance, the interplanar spacing of Pt‐Ce is 0.23 nm, corresponding to the (111) crystal facet of Pt_5_Ce intermetallic compound. The elemental distribution mappings of the Pt‐RE alloys highlight the good correspondence of Pt atoms with RE (Figure 2e), confirming the alloy composition. Overall, these results demonstrate that the RJTS method is universal for the controllable production of Pt‐RE alloys in a short time.

**Figure 3:**
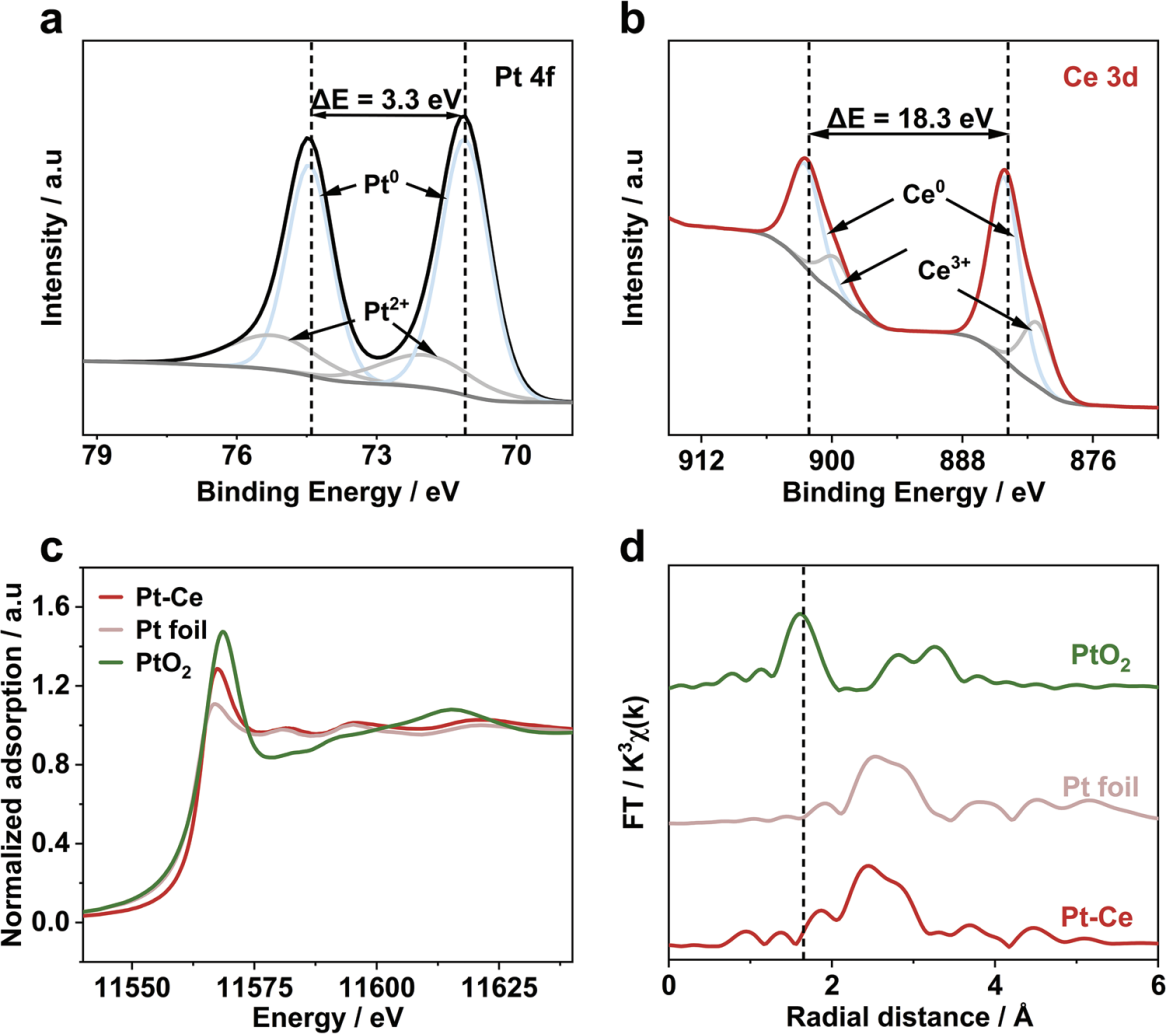
XPS spectra of a) Pt 4f, peaks corresponding to Pt 4f_5/2_ and Pt 4f_7/2_ of Pt (Pt^0^) appear at 74.4 and 71.1 eV.^[^ ^31^ ^]^ While the peak centered at 75.1 and 71.9 eV are described for Pt 4f_5/2_ and Pt 4f_7/2_ of the oxidized Pt (Pt^2+^), b) Ce 3d for Pt‐Ce‐80A‐100s catalyst. c) XANES and d) EXAFS spectra at Pt L3‐edge for Pt‐Ce‐80A‐100s, PtO_2_, and Pt foil.

The Pt 4f spectra shows the predominance of Pt^0^ in Pt‐RE alloy (**Figure** **3**a; Figure S14, Table S2, Supporting Information) and a ≈0.5 eV negative shift of the binding energy compared to Pt/C sample (Figure S15, Supporting Information), demonstrating the electron transfer from Ce to Pt.^[^ ^31^, ^36^ ^]^ Figure 3b displays spin doublets at 902.1 eV (Ce 3d_3/2_) and 883.8 eV (Ce 3d_5/2_), indicating that Ce mainly exists in Ce^0^ states.^[^ ^37^ ^]^ The other two weak peaks center at 899.4 eV (Ce 3d_3/2_) and 881.5 eV (Ce 3d_3/2_) are attributed to the transfer of electrons from Ce to Pt.^[^ ^29^ ^]^ As shown in Figure 3c, the white line peak of Pt‐Ce is similar to that of Pt foil but significantly lower than that of PtO_2_, indicating Pt^0^ in Pt‐Ce alloy, which is consistent with XPS results. In Figure 3d, a shortened Pt‐Pt bond length is observed compared to Pt foil. This indicates Pt exists in a metallic state in Pt‐Ce. These results confirm Ce has effectively regulating the electronic structure of Pt.^[^ ^38^ ^]^ The aforementioned evidence shows that Ce has a substantial regulatory effect on the electronic structure of Pt. This is achieved through the transfer of electrons from low electronegativity atoms (Ce) to high electronegativity atoms (Pt). As a result, the d‐band center of Pt shifts downwards, and the coordination bond length is shortened. These changes are beneficial for enhancing the catalytic performance of Pt in the ORR.

### 2.2 ORR Performance of Pt‐RE Catalysts

**Figure 4:**
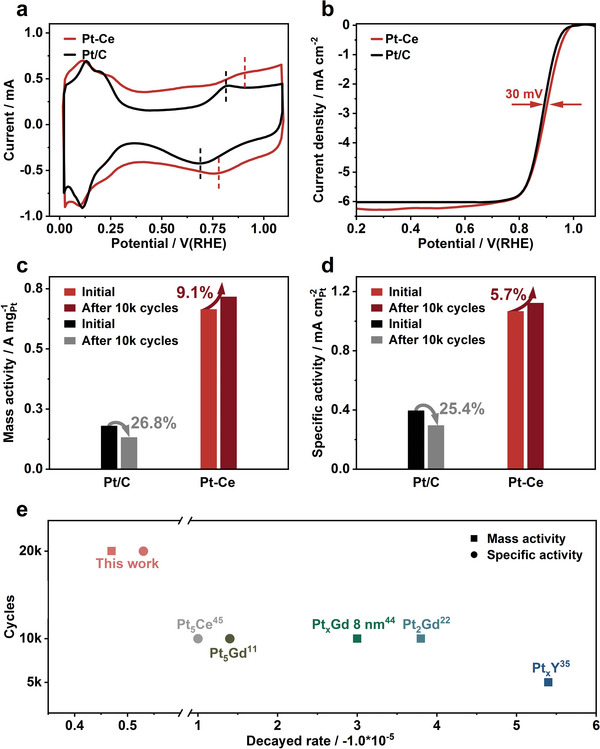
The ORR performance. a) CV curves of Pt‐Ce alloy and Pt/C catalysts in N_2_‐saturated 0.1 M HClO_4_ electrolyte. b) LSV curves of Pt‐Ce alloy and Pt/C catalysts obtained at 1600 rpm in 0.1 M HClO_4_ electrolyte. c) Mass activity and d) specific activity values of two catalysts obtained at 0.9 V before and after ADT test. e) Scatter plots comparing the mass activity and specific activity decay rate of different samples after different cycles.

We used a three‐electrode cell to investigate the ORR performance of Pt‐Ce alloy catalyst. The peaks in the Pt‐Ce alloy catalyst shift to higher potentials compared to commercial Pt/C catalyst (**Figure** **4**a), indicating excellent ORR activity of Pt‐Ce alloy catalyst. Specifically, the potential of the reduction peak is 0.77 V, which is 20 mV higher than Pt/C, while the potential of the oxidation peak is 0.92 V, 10 mV higher than Pt/C. This indicates that the bonding strength between Pt and oxygenated intermediates is weakened, which is because of the down shift of d‐band center of Pt by alloying with Ce.^[^ ^35^, ^39^, ^40^, ^41^, ^42^, ^43^ ^]^ In Figure 4b, the E_h_ of Pt‐Ce alloy is 0.91 V, which is higher than that of Pt/C catalyst (0.88 V). Interestingly, the catalytic performance of Pt‐Ce alloy shows a self‐optimization effect during the CV test, as shown in Figure 4c. Initially, Pt‐Ce alloy exhibits a high MA of 0.67 A mg_Pt_^−1^ at 0.9 V, which is 3.7 times higher than Pt/C (0.18 A mg_Pt_^−1^) and ≈1.5 times the DOE 2025 MA target. After ADT test with 10k cycles, the MA of Pt‐Ce increase by 9.1% to 0.72 A mg_Pt_^−1^. In contrast, the MA of Pt/C sharply decreased to 0.13 A mg_Pt_^−1^ with a 27.8% loss after 10k cycles. With regard to SA, the Pt‐Ce alloy demonstrates superior performance with a SA of 1.07 mA cm^−2^_Pt_ at 0.9 V, which is ≈2.7 times higher than Pt/C (0.39 mA cm^−2^_Pt_) (Figure 4d). After self‐optimization process, the SA of Pt‐Ce increased by 5.7% to 1.12 mA cm^−2^ which is still nearly 3.9 times higher than that of Pt/C. The stability advantage of Pt‐Ce alloy is illustrated in Figure 4e; Figures S16–S18, and Table S3 (Supporting Information), where shown significant superiority compared with reported literature. Notably, the decayed rate of MA or SA in this study is only ≈−0.5 × 10^−5^ per cycle, which is even one‐tenth of reported values.^[^ ^11^, ^22^, ^35^, ^44^, ^45^ ^]^ Overall, Pt‐Ce alloy with low Pt contents displays exceptional ORR activity and stability, making it a promising candidate for ORR.

**Figure 5:**
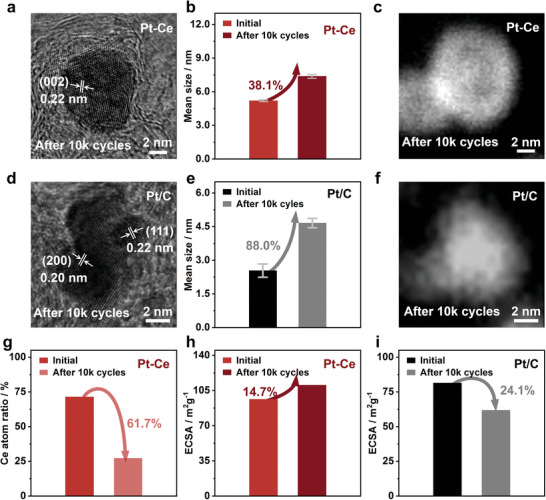
Stability investigation of two catalysts. a) The HR‐TEM image, b) histogram of particle size distribution, and c) HAADF‐STEM image of Pt‐Ce alloy after 10k cycles ADT test. d) The HR‐TEM image, e) histogram of particle size distribution, and f) HAADF‐STEM image of Pt/C after 10k cycles ADT test. g) The histogram of Ce atom ratio of Pt‐Ce alloy after 10k cycles ADT test. The histogram of ECSA value variation of h) Pt‐Ce alloy and i) Pt/C after 10k cycles ADT test.

The outstanding performance of Pt‐Ce alloy for ORR is primarily attributed to several factors. First, the positive electronic structure regulation of Ce in Pt‐Ce weakens the bonding of Pt with O, thereby promoting the desorption of oxygenated intermediates on the Pt site.^[^ ^23^ ^]^ The Pt‐Ce alloy is ≈5 nm, within the recognized particle size range of ORR catalysts. The Pt_5_Ce with a hexagonal structure exposes more Pt active sites.^[^ ^11^ ^]^ Additionally, the embedded alloy‐substrate structure further enhances the mechanically stability of the catalyst to avoid alloy aggregating and falling off. The negative alloying energy of Pt rare earth alloy increases its degradation resistance.^[^ ^6^ ^]^ To demonstrate the excellent stability of Pt‐Ce alloy catalyst, we conducted a characterization of the microscopic morphology and element contents of Pt‐Ce catalysts after 10k cycles. The results show that the alloys still maintain high quality of crystallinity with clear lattice fringes (**Figure** 5a). Furthermore, based on the TEM images in Figures S19–S21 (Supporting Information), the average particle size is found to be ≈7.5 nm with a 38.1% increase from Figure 5b, indicating that the embedded alloy‐substrate structure is robust and can effectively prevent particle aggregating. The HADDF image in Figure 5c confirms the stronger signals on the outer layer, indicating Pt skin formation, which is the primary reason for the improved performance of Pt‐Ce alloy.^[^ ^46^, ^47^ ^]^ In contrast, the HR‐TEM image of Pt/C (Figure 5d) reveals that the particles have undergone aggregation after ADT, resulting in a particle size that is almost twice as large as the original size observed in Figure 5e,f; Figures S22 and S23 (Supporting Information). XPS analysis of Pt‐Ce alloy after ADT (Figure S24, Supporting Information) indicates a 61.7% decrease in Ce on the surface, confirming the formation of Pt‐skin with more active sites on the alloy surface (Figure 5g; Table S4, Supporting Information). Moreover, comparing the ECSA values of the two samples in Figure 5h,i, we observe a 14.7% increase in Pt‐Ce alloy while a 24.1% decrease in Pt/C, providing direct evidence for the difference in ORR performance of the two catalysts. The DFT calculations results (Figure S25, Supporting Information) further demonstrate that the Pt‐Ce alloys in the intermetallic compound state can significantly enhance ORR performance. These results validate that our Pt‐RE alloys synthesized by RJTS method show robust ORR performance.

**Figure 6:**
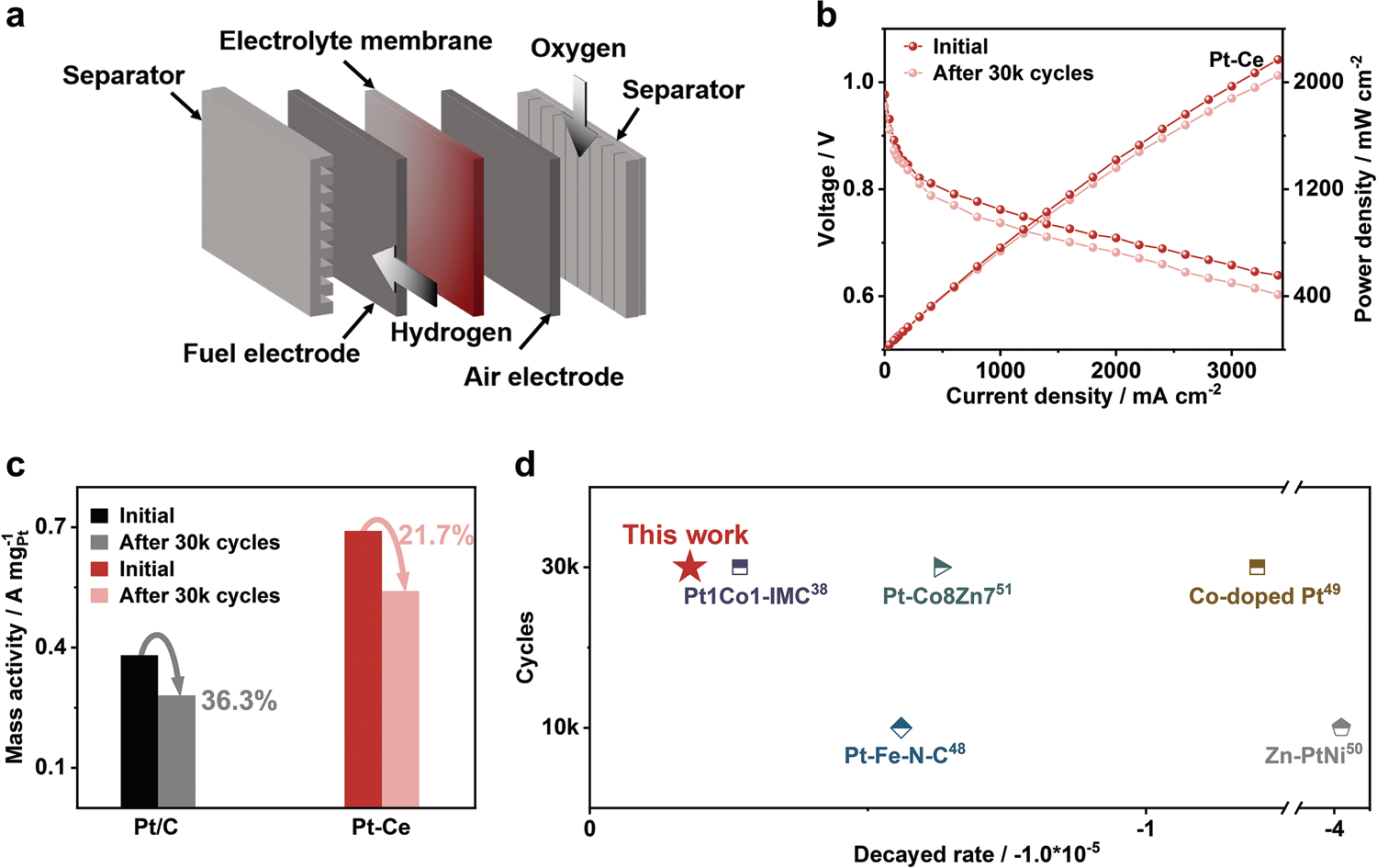
Practical performance of Pt‐Ce in PEMFC. a) The demo of practical application of Pt‐Ce in PEMFC. b) *I–V* polarization and power density curves of the H_2_‐O_2_ fuel cell with Pt‐Ce. c) Histogram of MA of Pt‐Ce and Pt/C. d) Scatter plots comparing the peak power density decayed rate of different samples after ADT tests.

We utilize the Pt‐Ce alloy as the cathodic catalyst in a PEMFC (**Figure** **6**a) and conduct a single‐cell test at a back‐pressure of 1.5 bar using an acidic H_2_/O_2_ PEMFC. As for operated in H_2_/Air, results are shown in Figure S26 (Supporting Information).The Pt loading of the membrane electrode assembly (MEA) utilizing Pt‐Ce or Pt/C at the cathode is 0.1 mg_Pt_ cm^−^2 Additionally, the Pt loading at anode is 0.1 mg_Pt_ cm^−^2 The fuel cell utilizing Pt‐Ce alloy at the cathode exhibits a current density of 3.06 A cm^−^2 at 0.65 V and a peak power density of 2.17 W cm^−^2 (Figure 6b). The above power density accounts for nearly two times than the commercial Pt/C‐based MEA (Figure S27, Supporting Information). After the stability test of 30k cycles, the peak power density shows a small decrease of 5.4% in Pt‐Ce alloy assembled PEMFC. More clearly, Figure 6c shows that the MA of Pt‐Ce in the PEMFC is remarkable, at 0.69 A mg_Pt_^−1^ at 0.9 V, representing 3.1 times higher than that of the Pt/C and 1.57 times higher than that of the DOE 2025 activity target (0.44 A mg_Pt_^−1^). And it only decreased 21.7% to 0.54 A mg_Pt_^−1^ after ADT, while Pt/C decreased 36.4%. The stability advantage of Pt‐Ce alloy in PEMFC is also worthy mentioned as shown in Figure 6d; Table S5 (Supporting Information), where shown significant superiority compared with reported Pt‐based catalysts.^[^ ^38^, ^48^, ^49^, ^50^, ^51^ ^]^ As for the peak power density decayed rate, it values −0.5 × 10^−5^ per cycle, which is even one‐twenty of reported. Overall, the Pt‐Ce alloy catalyst exhibit excellent performance in PEMFC.

## 3 Conclusion

We have developed a RJTS method to prepare a family of Pt‐RE alloys. The RJTS method shows significant advantages of low energy consumption, and fast one‐step in the synthesis of Pt‐RE alloy family. The as‐synthesized Pt‐RE alloys show uniform size distribution and high lattice quality. The Pt‐RE alloy electrocatalysts show superior ORR performance. For example, both the MA and SA of the Pt‐Ce alloy catalyst are 3.7 times and 2.7 times higher than that of the Pt/C catalyst. Furthermore, the performance of Pt‐Ce alloy shows negligible decay after ADT test with 20k cycles, which thanks to high utilization of Pt sites and the strong anchoring of the substrate on the alloys. Its performance in PEMFC exhibits excellent stability with an ultralow decayed rate of peak power density −0.5 × 10^−5^ and MA decrease rate of −5 × 10^−6^ A mgPt^−1^ per cycle after 30k cycles. This RJTS method can synthesis binary alloys or high entropy alloys beyond Pt‐RE alloys by simple process and low energy cost, paving a way to prepare noble/non‐noble metal alloys for widely catalytic applications.

## Conflict of Interest

The authors declare no conflict of interest.

## Supporting information

Supporting Information

## Data Availability

The data that support the findings of this study are available on request from the corresponding author. The data are not publicly available due to privacy or ethical restrictions.
