# Supplementary material for: Universal and Energy‐Efficient Approach to Synthesize Pt‐Rare Earth Metal Alloys for Proton Exchange Membrane Fuel Cell
Source: Adv Sci (Weinh). 2023 Nov 20;11(1):2305110. doi: 10.1002/advs.202305110 (PMC10767455; doi:10.1002/advs.202305110)
Supplement: Supplementary file 1 — Supporting Information [file ADVS-11-2305110-s001.pdf]

ADVANCED SCIENCE
Open Access

Supporting Information

for *Adv. Sci.*, DOI 10.1002/advs.202305110

Universal and Energy-Efficient Approach to Synthesize Pt-Rare Earth Metal Alloys for Proton Exchange Membrane Fuel Cell

Nannan Jiang, Bing Huang, Minghao Wang, Yumo Chen, Qiangmin Yu and Lunhui Guan**

Supporting Information

Universal and energy-efficient approach to synthesize Pt-rare earth metal alloys for proton exchange membrane fuel cell

Nannan Jiang[1,3], Bing Huang[1,3], Minghao Wang[1], Yumo Chen[2], Qiangmin Yu[2,], and Lunhui Guan[1,*]*

1. CAS Key Laboratory of Design and Assembly of Functional Nanostructures & Fujian Key Laboratory of Nanomaterials, Fujian Institute of Research on the Structure of Matter, Chinese Academy of Sciences, Fuzhou, 350000, China

2. Shenzhen Geim Graphene Center, Tsinghua-Berkeley Shenzhen Institute & Institute of Materials Research, Tsinghua Shenzhen International Graduate School, Tsinghua University, Shenzhen 518055, P.R. China

3. University of Chinese Academy of Sciences, Beijing, 100049, China

Corresponding authors

Qiangmin Yu, e-mail: yu.qiangmin@sz.tsinghua.edu.cn

Lunhui Guan, e-mail: guanlh@fjirsm.ac.cn

Experimental Section

Catalyst synthesis. A certain amount of chloroplatinic acid, rare earth metal salt, g-C_3N_4 and N-KJB were grinded in the ambient atmosphere and mixed evenly in an agate mortar. N-KJB was synthesized by melamine and KJB treated in Ar at 700 ºC for 30

min. Putting urea in muffle furnace in air at 550 °C for 3 h is to obtain g-C_3N_4. First, the g-C_3N_4 as ligand according to the reported method, and nitrogen doped Ketjen Black EC600JD with a high specific surface area of 1164.2 m^2/g (**Figures S28-S29**) was used as support to uniformly disperse the metal salts. The detailed feeding mass of the reactants are presented as Table S6 The mixtures were filled into a high-purity graphite rod, loaded into the electric arc furnace, later pyrolyzed by general thermal treatment at different current from 70-100 A for 20-120 s. With the utilization of an infrared temperature sounder, we are able to obtain real-time temperature measurements. Subsequently, we can easily regulate the temperature by controlling the current, ensuring convenient operational control. Furthermore, the timing mechanism enables us to terminate the powder supply whenever necessary. Then, the obtained materials were treated in a beaker with 1 mol L^{-1} H_2SO_4 at 60 °C for 1 h. Additionally, after filtration, the as-synthesized Pt-RE alloy is basically wrapped by thin carbon shell (**Figure S30**), so it needed to be removed and put in the air at 200 °C for 12 h by mild oxidation removal. And obtained a family of Pt-RE with the 8wt% loading capacity of Pt on N-KJB. We adjusted the atom ratio of Pt and RE to achieve the optimal atom ratio of 1:2. Taking Pt-Ce alloy as typical example, when the atom ratio of Pt: Ce was 2:1, the obtained XRD pattern implied that only the diffraction peak of Pt (**Figure S31**) appeared. When the ratio of Pt: Ce decreased to 1:4, the alloy phase was complex, containing several phases of Pt-Ce binary alloys (**Figure S32**). Besides, Pt-Gd treated in 80 A but different time (80 s, 100 s, and 120 s) also showed the same law compared with Pt-Ce treated in 80 A (80 s, 100 s, and 120 s), the details shown in **Figures S33-**

S38.

Physical characterization. Powder X-ray diffraction (XRD) measurements were performed with a MiniFlex 600 X-ray diffractometer (Rigaku) using a Cu kα (λ= 1.5418 Å) radiation source with 30 kV and 15 mA. The scan speed was 1° per minute. The Scherrer formula: $D = K * \lambda / (\beta * \cos\theta)$ was used to calculate the grain size *D*, the average grain size is D, the crystal shape factor K values 0.9, the diffraction angle θ and semi-peak width β, respectively. Inductively coupled plasma-optical emission spectroscopy (ICP-OES) analyses were performed in Agilent ICP-OES 720. The results based on quantitative inductively coupled plasma-optical emission spectroscopy (ICP-OES) analysis (Table S7) indicated that the molar ratio of Pt to RE in the alloy were nearly similar. Based on the above results, Pt−RE alloys with the Pt loading of about 9 wt% on the N-doped carbon support were obtained following this synthesis route. X-ray photoelectron spectrometry (XPS) was carried out on a Thermo Scientific K-Alpha+ X-ray photoelectron spectrometer with an Al Kα X-ray monochromator. The auger spectrum of Ce MNN is shown in **Figure S39**, further confirming the metallic state of Ce.[1] Transmission electron microscope (TEM) images were obtained at 200 kV with a Talos-F200X and Tecnai G^2 F20. And the high-angle annular dark-field scanning transmission electron microscopy (HAADF-STEM) images were obtained with a Talos-F200X at 200 kV. The inner morphology of the as-synthesized samples was characterized by transmission electron microscopy. High-resolution (HR)-TEM used to analyze the morphology of samples. More than 300 particles from 3 to 5 TEM images

were counted for statistics to determine the particle size distribution of each sample. The elemental mappings were acquired by EDS characterization. In additionally, we also investigated the element composition from different particles (**Figure S40**), the results show that Pt and Re are well-dispersed in each particle. The AC-TEM image was obtained by JEM-ARM300F. The specific surface area of carbon support N-KJB were measured using the Brunauer-Emmett-Teller (BET) surface area analyzer (Autosorb IQ).

Energy consumption calculation. We use electric arc furnace (Panasonic, YD – 400SS) to synthesize. As it shown in the machine's instruction, "60 A / 22.4 V ~ 400 A / 36 V", there is a linear relationship between voltage and current. Taking Pt-Ce-80A-100s as an example, when the current is 80 A, voltage values 23.2 V. We used to calculate the energy consumption by eq 1 and 2.

$$W = P \cdot t \quad \text{(eq 1)}$$

Where P is the power, t is the synthesis time.

$$P = U \cdot I \quad \text{(eq 2)}$$

Where U is the output voltage, I is the current.

So, during the 100 s, energy consumption W is about 0.052 kW·h.

In the calculation process, the processing time refers to the sum of the heating time and holding time. When calculating the energy consumption required for the process reported in the references, the working parameters of the OTF-1200X1200 Celsius opening tube furnace from Hefei Kejing Company were used for calculations, and the

power is 3 kW.

The thermal consumption Q is calculated by eq R3 and the energy efficiency η is calculated by eq R4.

$$\mathrm{Q = M \cdot C \cdot \Delta T} \quad \text{(eq 3)}$$

Where M is the mass of graphite rod, C is the specific heat capacity and ΔT is the temperature difference before and after reaction.

$$\eta = \frac{P_O}{P_I} = \frac{W-Q}{W} \quad \text{(eq 4)}$$

Where P_O is output power, P_I is input power, W is the total energy consumption, and Q is thermal consumption.

Take Pt-Ce-80A-100s as an example. The average mass of graphite rod is 2.5×10^{-3} kg, C is 710 J/(kg·K), and ΔT is nearly 1000 K, thus Q values 1775 J, equals 4.93×10^{-4} kW·h. While the total energy consumption is about 0.052 kW·h, thus η values nearly 99.1%.

Electrochemical measurements. In order to assess the ORR performance, a typical three-electrode configuration with rotating disk electrode (RDE) setup was applied. A Pt foil was applied as the counter electrode, and a commercial Ag/AgCl was used as the reference electrode. 5 mg of the catalysts were added to 1 mL of solution consisted of Nafion solution (5 wt%) and iso-propanol. The Pt loading of Pt-RE and the commercial Pt/C (20 wt %, Johnson-Matthey Co.) on the glassy carbon electrode (0.196 cm^2) was kept at 8.9 $\mu g_{Pt}/cm^2$. All electrochemical measurements were performed in 0.1 M $HClO_4$ at 25 °C. Cyclic voltammetry (CV) measurement was conducted in O_2-saturated

0.1 M $HClO_4$ for at least 60 cycles. Secondly, the linear sweep voltammetry (LSV) curves were collected at 10 mV/s to evaluate the ORR activity, and the curves were calibrated with the background subtraction and solution resistance. According to the Koutecky-Levich equation (eq 5), the kinetic currents could be calculated. The mass activity (MA) could be calculated according to eq 6. And as for eq 7, the specific activity (SA) could be calculated.

$$\frac{1}{i} = \frac{1}{i_L} + \frac{1}{i_K} \qquad \text{(eq 5)}$$

where i is the measured current and i_L is the diffusion-limited current.

$$MA = \frac{i_K}{m_{Pt}} \qquad \text{(eq 6)}$$

where m_{Pt} is the Pt loading of the catalyst used in the working electrode.

$$SA = \frac{i_K}{ECSA} \qquad \text{(eq 7)}$$

where ECSA is the electrochemical surface area of the catalyst, which can be estimated by a hydrogen underpotential deposition (Hupd) method. Besides, to assess the ECSA, the Hupd method (assuming factor = 210 μC cm^{-2}) was applied. The Hupd method was conducted in 0.1 M $HClO_4$ saturated with N_2 (100 mV/s). In the potential range of around 0.011 V and 0.406 V after the deduction of the capacitive current, which contain the Hupd adsorption/desorption peak, and the charge (Q_H) was obtained. The baseline used for integration of the Q_H belonging to the Hupd adsorption/desorption peak was the current value at around 0.406 V. According to the eq 8, the ECSA obtained via the Hupd method was estimated.

$$ECSA = \frac{Q_H}{210 \times m_{Pt}} \quad \text{(eq 8)}$$

where m_{Pt} is the Pt loading of the as-prepared catalyst used in the working electrode. The average charge of the H adsorption and desorption peak was applied to estimate the ECSA (denoted as $\bar{H}s$) for comparison. At last, the stability of the catalysts was assessed by using an accelerated durability test (ADT) for 10k and 20k cycles at 0.6-1.0 V (vs RHE, 100 mV/s) in the O_2 atmosphere.

PEMFC measurement. The catalyst powder, Nafion (5wt%, Dupont) and isopropanol were ultrasonically mixed in a mass ratio of 1:X:100 to prepare the catalyst ink (X=12.5 for Pt-Ce, X=5 for 20 wt% JM Pt/C). The JM Pt/C cathode and Pt-Ce cathode are prepared by spraying the correspond catalyst ink on the gas diffusion layer (GDL, Sigracet 25 BC), the Pt loading is controlled within 0.1 mg_{Pt} cm^{-2}, respectively. All the anode of MEAs were prepared by spraying the JM Pt/C on the GDL (Sigracet 25 BC) with a Pt loading of 0.1 mg_{Pt} cm^{-2}. The MEAs with an active area of 25 cm^2 were fabricated by hot-pressing the prepared anode and cathode onto the opposite sides of a Nafion-117 membrane at 80 °C for 3 min under a pressure of 3 MPa. The MEA performance of the catalysts as the cathode was tested at a fuel cell testing system (Scribner 850e). The backpressure was set at 150 kPa for both the anode and cathode at H_2-O_2 and H_2-Air. For all fuel cell tests, the cell temperature was kept at 80 °C. Fully humidified hydrogen (flow rates: 600 sccm) and air (flow rates: 1500 sccm) at 80 °C were supplied to the anode and cathode, respectively. A chronoamperometric test at a constant voltage of 0.6 V and at 80 °C in H_2(anode)-O_2(cathode) was adopted to

evaluate the stability of Pt-Ce alloy catalyst. We also applied Nafion 211 to PEMFC devices and conducted performance tests on Pt-Ce and commercial Pt/C using the same test process. The results are shown in the **Figure S41**.

Density functional theory (DFT) calculations. All the DFT computations were conducted employing the Vienna ab initio simulation package (VASP). The exchange-correlation energy was assessed utilizing the generalized gradient approximation (GGA) with the Perdew–Burke–Ernzerhof (PBE) functional, and the projector augmented wave (PAW) method was employed to model electron-ion interactions. The energy cutoff for plane wave expansions was set at 450 eV, with a convergence threshold established at 10^{-5} eV in energy and 0.03 eV $Å^{-1}$ in force, respectively. For slab model calculations, a vacuum space of 15 Å was introduced to prevent interactions between periodic images. Furthermore, a dipolar correction was applied to slab models with symmetrization switched off. K-POINT was set to 2 × 2 × 1.

For intermetallic compounds, we constructed the structure of Pt_5Ce (1 1 1) crystal facet, comprising 4 layers. For solid solution structure, we employed the Pt (1 1 1) crystal facet, randomly arrange Pt and Ce atoms, also containing 4 layers. Both structures encompassed 16 Ce atoms and 80 Pt atoms, with the lower three layers firmly held during the computational process. To evaluate the ORR performance for slab models, we adopted the typical adsorbate evolution mechanism as proposed by Nørskov et al.[2,3]

Table S1. Different synthetic conditions of preparing Pt-RE alloys reported recent years.

method	Pt-RE	Time	Gas atmosphere	References
Physical Metallurgical	Dy–Pt, Ho–Pt		0.7 bar argon	*J. Alloys Compd.* **2005**, *391*, 60[4]

Method	Sc-Pt, Y-Pt		Vacuum	*Top. Catal.* **2013**, *57*, 245[5]
Magnetron Sputtering Method	Pt-La	dual-gun magnetron co-sputtering system	Ultrahigh vacuum	*Energy Environ. Sci.* **2012**, *5*, 7521[6]
	Pt-Gd	gas aggregation method and filtered by a time-of-flight mass filter	Ultrahigh vacuum	*J. Catal.* **2015**, *328*, 297[7]
Electrodeposition Method	Pt-Pr	the reverse process of electrodepositio n and electro-eroded electrodes		*ACS Appl. Mater. Interfaces.* **2019**, *11*, 5129[8]
Chemical Reduction Method	Pt-Y	6h<br>at least 80 min	Hydrogen	*ACS Catal.* **2018**, *8*, 2071[9]
	Pt-Gd, Pt-Ce, Pt-La, Pt-Tb, Pt-Sm, Pt-Y	255 min	7% H_2/Ar	*J. Am. Chem. Soc.* **2020**, *142*, 953[10]
**Rapid Joule thermal-shock (RJTS)**	**Pt-Ce, Pt-Gd, Pt-La, Pt-Tb, Pt-Sm, Pt-Y**	**100 s**	**10 Pa vacumm**	**This work**

Table S2. The synthesis time and energy consumption compared with peers.

Sample	Synthesis time / s	Energy consumption / Kw h	Reference
Pt_5Ce	14250	9.875	*J. Am. Chem. Soc.* **2020**, *142*, 953[10]
Pt-La	9000	6.250	*Chem. Mate*r. **2022**, 34, 10789[11]

Pt_xY	10950	7.604	*ACS Appl. Energy Mater.* **2022**, 5, 3319[12]
Pt_xRE/C	14100	9.790	*RSC Adv.* **2022**, 12, 4805[13]
Pt-Ln	15750	10.938	*Energy Environ. Sci.* **2021**, 14, 5911[14]
**Pt-Ce**	**100**	**0.052**	**This work**

Table S3. The electrochemical tests of Pt-RE reported recent years.

Sample	Catalytic activities at 0.9 V (*vs*. RHE)		Improvement factor(a)	Improvement factor(b)	After 10k cycles		References
	MA(A/mg_{Pt})	SA (mA/ cm^2 $_{Pt}$)			SA (mA/cm^2 $_{Pt}$)	Improvement factor(c)	
Pt_xPr/C	0.70	1.96	1.7	N/A	N/A	N/A	*ACS Appl. Mater. Interfaces.* **2019**, *11*, 5129[8]
A-Pt-Gd(5/1)	0.35	0.50	N/A	N/A	N/A	N/A	J. *Phys. Chem. C.* **2020**, *124*, 26925[15]
Pt_5Gd	N/A	10.4	N/A	5	-14%	N/A	*J. Am. Chem. Soc.* **2012**, *134*, 16476[16]
Pt_5Ce(3.5nm)	~ 0.70	1.16	1.4	1.5	N/A	N/A	*J. Am. Chem. Soc.* **2020**, *142*, 953[10]
Pt_5Ce(5.7nm)	~ 0.95	2.29	1.9	3.1			
Pt_5Ce(8.8nm)	~ 0.80	3.95	1.6	5.3			
Pt_xGd(5nm)	3.10	N/A	3.1	N/A	-47.7%	N/A	*J. Catal.* **2015**, *328*, 297[7]
Pt_xGd(8nm)	3.60		3.6		-32.5%		
Pt_xGd(9nm)	2.40		2.4		-54.2%		
Pt_5La	N/A	~ 6.8	N/A	3.4	-8%	N/A	*J. Mater. Chem. A.* **2014**, *2*, 4234[17]
Pt_5Ce		~ 7.4		3.7	-10%		
Pt_5Gd		~ 10.4		5.2	N/A		

Pt_5Gd(8 nm)	~3.30	10.5	13.2	5.2	9.1	4.7	*Science.* **2016,** *352*, 73[18]
Pt_5Ce	N/A	7.4	N/A	3.5	6.6	3.4	
**Pt-Ce**	**0.7**	**1.1**	**3.7**	**2.7**	**1.1**	**3.9**	**This work**

(a) Improvement factor was determined by MA (sample)/MA(Pt) in the literature.

(b) Improvement factor was determined by SA (sample)/SA(Pt) in the literature.

(c) Improvement factor was determined by after ADT SA (sample)/SA(Pt) in the literature.

Table S4. The bulk elemental compositions of samples determined by XPS.

Sample	Synthesis conditions	Pt / at%	Rare-earth metal / at%	Pt/RE molar ratio
Pt-Ce	After 10k cycles	58.3	41.7	1.40
	After 10k cycles	48.1	51.9	0.93

Table S5. The PEMFC performance of Pt-based alloy catalysts reported recent years.

Sample	Current density (A/cm^2)	Peak power density (W/cm^2)	MA (A/mg_{Pt})	After 30k cycles Peak power density decayed (%)	After 30k cycles MA decayed (%)	References
Pt Fe/ Fe-N-C (H_2/O_2,1.5 bar)	2.9 (0.6 V)	2.3 (4.2 A/cm^2)	N/A	N/A	N/A	*Adv. Mater.* **2023**, e2300624[19]
PIFCC-HEI/C (H_2/O_2,1 bar)	4.25 (0.4 V)	1.73	~0.1	N/A	N/A	*J. Am. Chem. Soc.* **2023**[20]
Pt-Fe-N-C (H_2/O_2, 1bar)	~1.4 (0.65 V) ~0.05 (0.9V)	1.08 (2.0 A/cm^2)	0.77	~5.6 (10k)	N/A	*Nat. Catal.* **2022**, 5, 503[21]
Pt1Co1-IMC@Pt/C (H_2/O_2 1 bar)	~0.28 (0.8 V)	2.30 (4.0 A/cm^2)	0.18	8.0	24.8	*Energy Environ. Sci.*, **2022**, 15, 278[22]
Ce SAS/HPNC (H_2/O_2, 2 bar)	0.47 (0.6 V)	0.49	0.52	N/A	N/A	*ACS Catal.* **2021**, 11, 3923[23]

PtCo i-NPs (H_2/O_2 1.5 bar)	~0.3 (0.8 V)	1.2	1.52	N/A	~21	*Science* **2021**, 374, 459[24]
Co-doped Pt/C (H_2/Air 1.5 bar)	~0.3 (0.8 V)	1.1	~0.25	36.4	~75	*Adv. Energy Mater.* **2022**, 12, 2103144[25]
Zn-PtNi/C (H_2/O_2 1.5 bar)	~1.6 (0.6 V)	1.3	~0.2	~41 (10k)	N/A	*Adv. Funct. Mater.* **2022**, 33, 2212442[26]
PCNMC_Co8Zn7 (H_2/O_2 1.5 bar)	~0.5 (0.8V)	1.5	0.75	19	29	*Adv. Energy Mater.* **2022**, 12, 2201600[27]
PtCe/NC (H_2/O_2)	~0.4 (0.6 V)	0.61	~0.12	31.6	N/A	*Chem. Asian J.* **2023**, 18, e202300137[28]
**Pt-Ce (H_2/Air 1.5bar)**	**2.12 (0.65 V), 0.43 (0.8 V), 0.03 (0.9 V)**	**1.62 (3 A/cm^2)**	**0.3**			**This work**
**Pt-Ce (H_2/O_2 1.5bar)**	**3.0 (0.65 V), 0.58 (0.8 V), 0.07 (0.9 V)**	**2.2 (3.4 A/cm^2)**	**0.69**	**5.6**	**21.7**	**This work**

Table S6. The amounts of precursors and the molar ratio of Pt particles and rare-earth metals.

Sample	$H_2PtCl_6\cdot 6H_2O$ mass / g	$RECl_x\cdot nH_2O$ mass / g	g-C_3N_4 mass / g	N-KJB mass / g	Pt/RE molar ratio
Pt-Ce		$CeCl_3\cdot 7H_2O$(99.99%)			
	0.1035	0.1490	0.04	0.1685	0.5
	0.1035	0.2980	0.04	0.1685	0.25
	0.4140	0.1490	0.04	0.1685	2
Pt-Gd	0.1035	$GdCl_3\cdot 6H_2O$(99%+) 0.1487	0.04	0.1685	0.5
Pt-La	0.1035	$LaCl_3\cdot xH_2O$(99%+) 0.0981	0.04	0.1685	0.5
Pt-Sm	0.1035	$SmCl_3\cdot 6H_2O$(98%+) 0.1459	0.04	0.1685	0.5
Pt-Tb	0.1035	$TbCl_3\cdot 6H_2O$(99.99%)	0.04	0.1685	0.5

		0.1494			
Pt-Y	0.1035	$YCl_3 \cdot 6H_2O$(99.99%) 0.1213	0.04	0.1685	0.5

$H_2PtCl_6 \cdot 6H_2O$ and $RECl_x \cdot 6H_2O$ were purchased from Tansoole.

Table S7. The bulk elemental compositions of samples determined by ICP and XPS.

Sample	Determined by ICP			Determined by XPS		
	Pt / wt%	Rare-earth metals / wt%	Pt/RE molar ratio	Pt / at%	Rare-earth metals / at%	Pt/RE molar ratio
Pt-Ce	8.6	11.4	0.54	28.6	71.4	0.40
Pt-Gd	9.2	14.6	0.52	23.4	76.6	0.31
Pt-La	8.7	11.3	0.54	34.0	66.0	0.52
Pt-Sm	9.4	13.9	0.52	36.0	64.0	0.56
Pt-Tb	7.4	9.8	0.61	48.8	51.2	0.95
Pt-Y	8.1	12.5	0.3	41.8	58.2	0.72

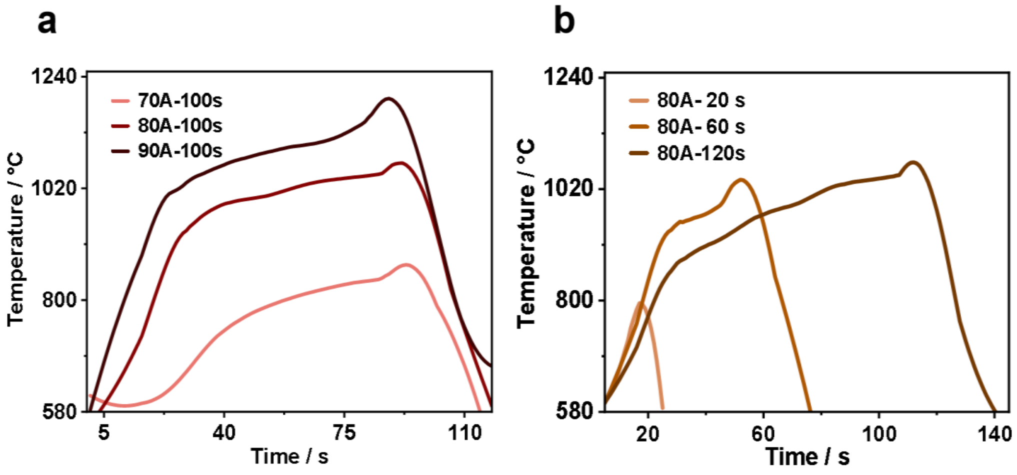

Figure S1. The temperatures measured by the real-time infrared thermometer at (a) the same time and (b) the same current.

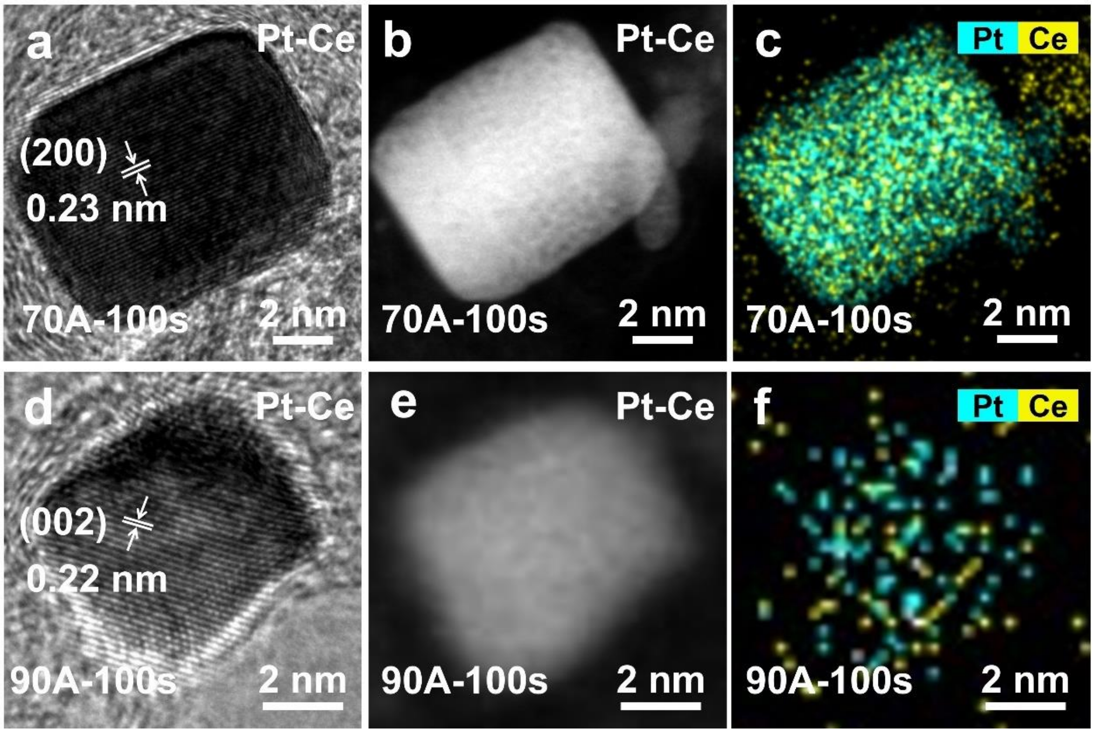

Figure S2. (a) The HR-TEM image of Pt-Ce-70A-100s. (b) The HAADF-STEM image of Pt-Ce-70A-100s. (c) The HAADF-STEM elements mapping of Pt-Ce-70A-100s. (d) The HR-TEM image of Pt-Ce-90A-100s. (e) The HAADF-STEM image of Pt-Ce-90A-

100s. (f) The HAADF-STEM elements mapping of Pt-Ce-90A-100s.

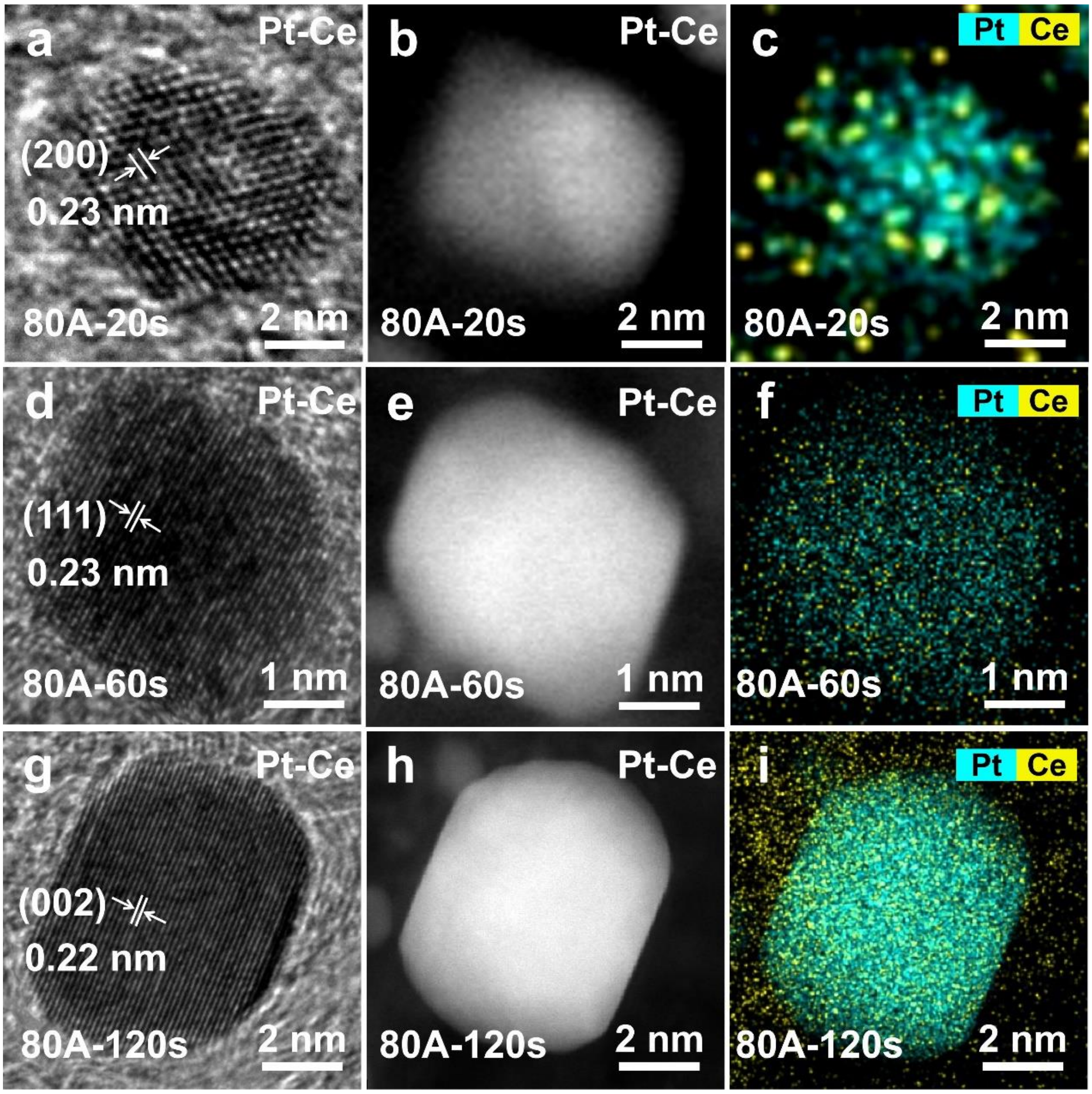

Figure S3. (a) The HR-TEM image of Pt-Ce-80A-20s. (b) The HAADF-STEM image of Pt-Ce-80A-20s. (c) The HAADF-STEM elements mapping of Pt-Ce-80A-20s. (d) The HR-TEM image of Pt-Ce-80A-60s. (e) The HAADF-STEM image of Pt-Ce-80A-60s. (f) The HAADF-STEM elements mapping of Pt-Ce-80A-60s. (g) The HR-TEM image of Pt-Ce-80A-120s. (h) The HAADF-STEM image of Pt-Ce-80A-120s. (i) The

HAADF-STEM elements mapping of Pt-Ce-80A-120s.

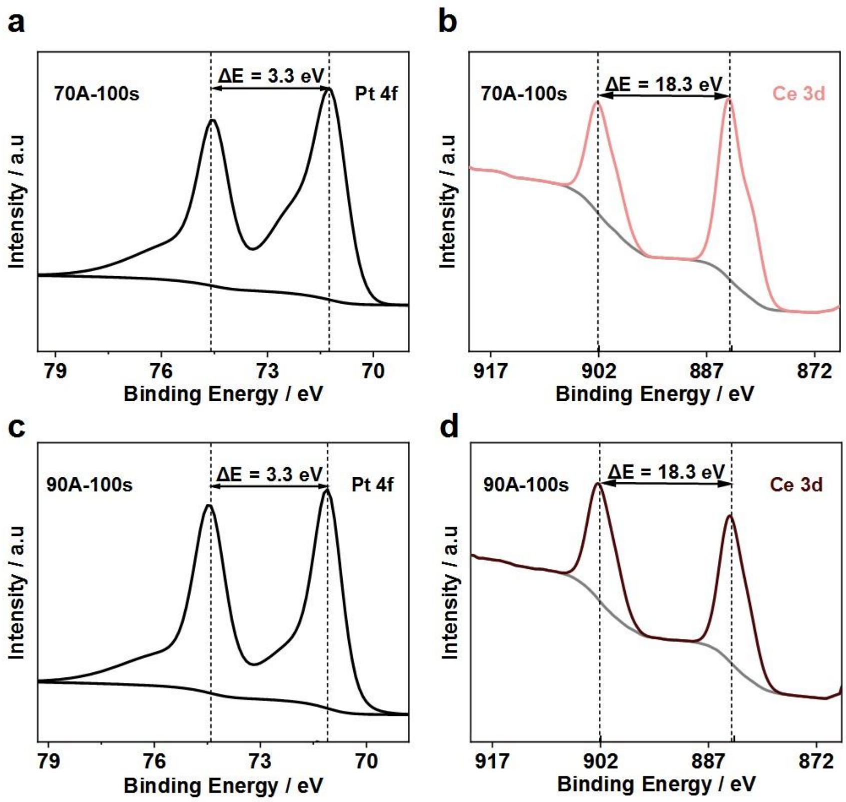

Figure S4. (a) The XPS Pt 4f spectrum of Pt-Ce-70A-100s (peaks corresponding to Pt (Pt^0) appear at 74.6 and 71.3 eV. (b) The XPS Ce 3d spectrum of Pt-Ce-70A-100s. (c) The XPS Pt 4f spectrum of Pt-Ce-90A-100s (peaks corresponding to Pt (Pt^0) appear at

74.6 and 71.3 eV. (d) The XPS Ce 3d spectrum of Pt-Ce-90A-100s.

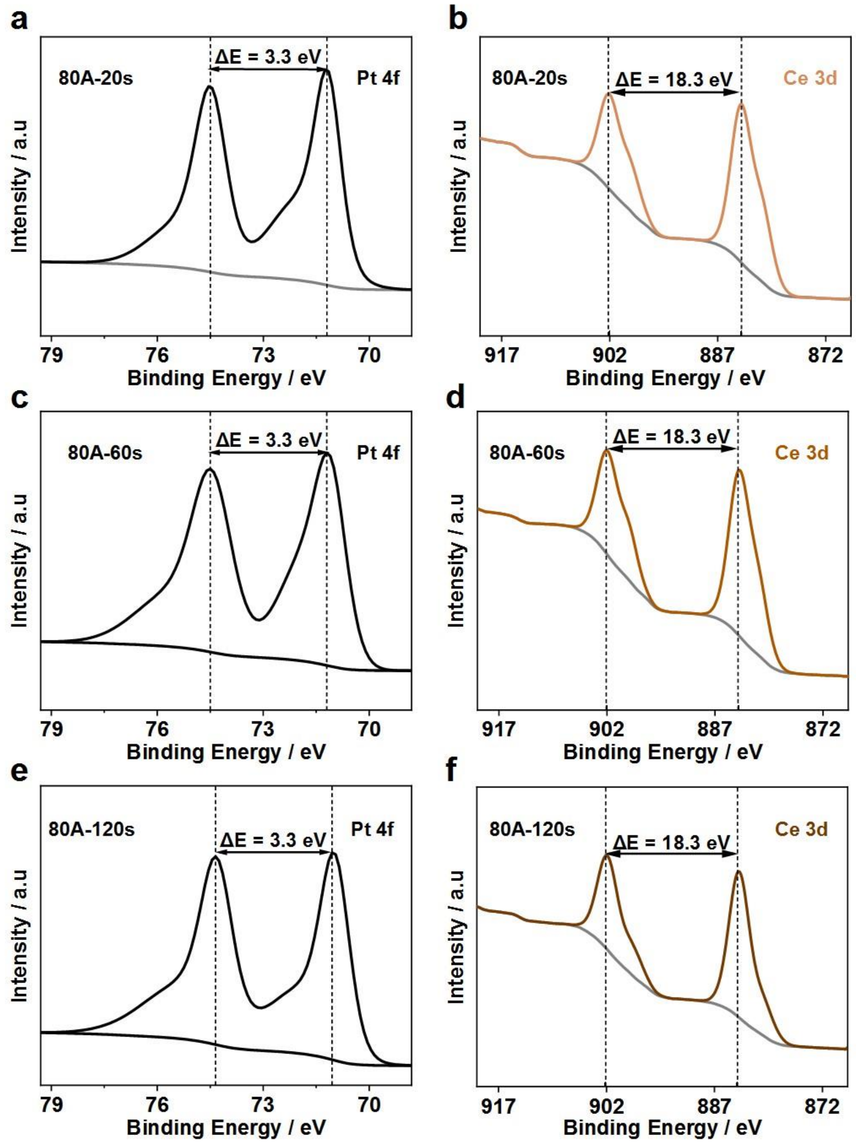

Figure S5. (a) The XPS Pt 4f spectrum of Pt-Ce-80A-20s (peaks corresponding to Pt (Pt^0) appear at 74.5 and 71.2 eV. (b) The XPS Ce 3d spectrum of Pt-Ce-80A-20s. (c) The XPS Pt 4f spectrum of Pt-Ce-80A-60s (peaks corresponding to Pt (Pt^0) appear at 74.5 and 71.2 eV. (d) The XPS Ce 3d spectrum of Pt-Ce-80A-60s. (e) The XPS Pt 4f spectrum of Pt-Ce-80A-120s (peaks corresponding to Pt (Pt^0) appear at 74.4 and 71.1

eV. (f) The XPS Ce 3d spectrum of Pt-Ce-80A-120s.

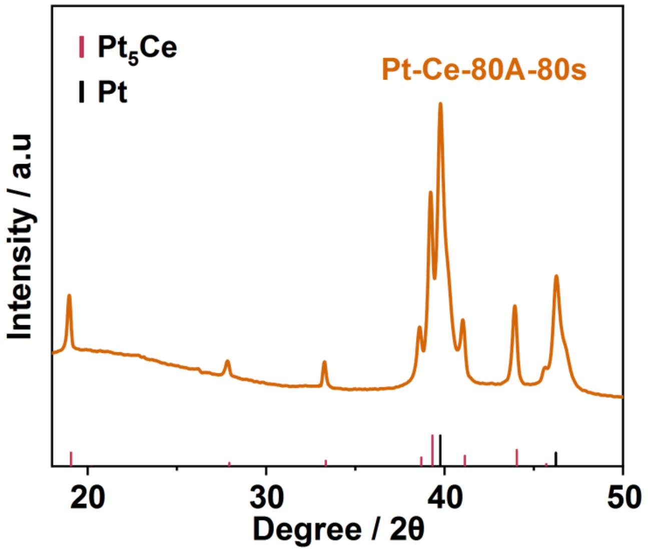

Figure S6. The XRD pattern of Pt-Ce-80A-80s.

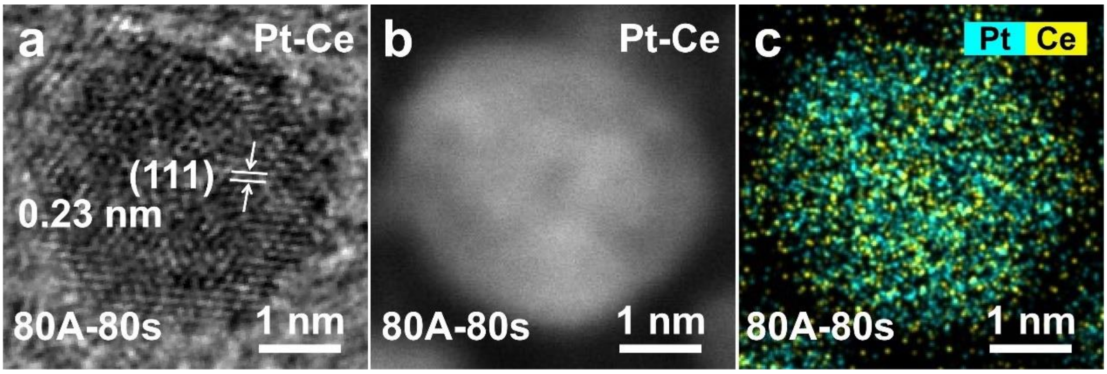

Figure S7. (a) The HR-TEM image of Pt-Ce-80A-80s. (b) The HAADF-STEM image of Pt-Ce-80A-80s. (c) The HAADF-STEM elements mapping.

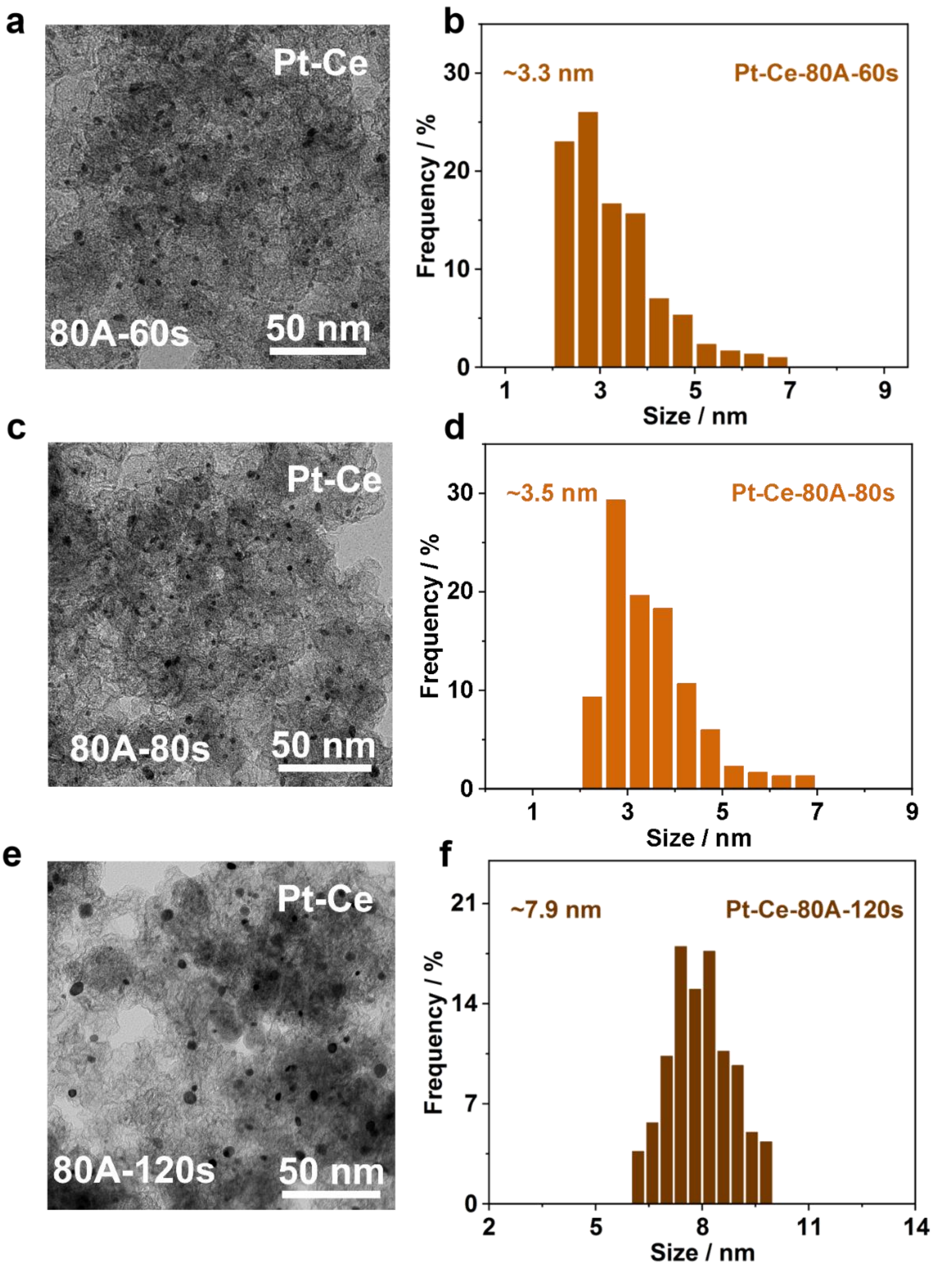

Figure S8. (a) The TEM image of Pt-Ce-80A-60s. (b) The histogram of particle size distribution of Pt-Ce-80A-60s. (c) The TEM image of Pt-Ce-80A-80s. (d) The histogram of particle size distribution of Pt-Ce-80A-80s. (e) The TEM image of Pt-Ce-80A-120s. (f) The histogram of particle size distribution of Pt-Ce-80A-120s.

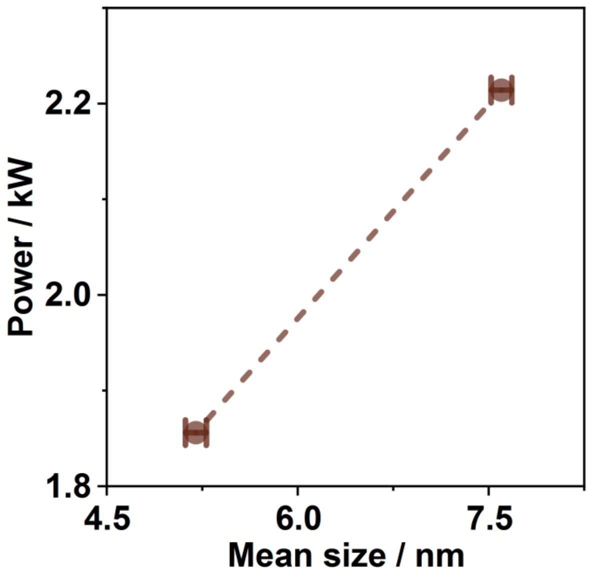

Figure S9. Dot line diagram of the relationship between mean particle sizes and power.

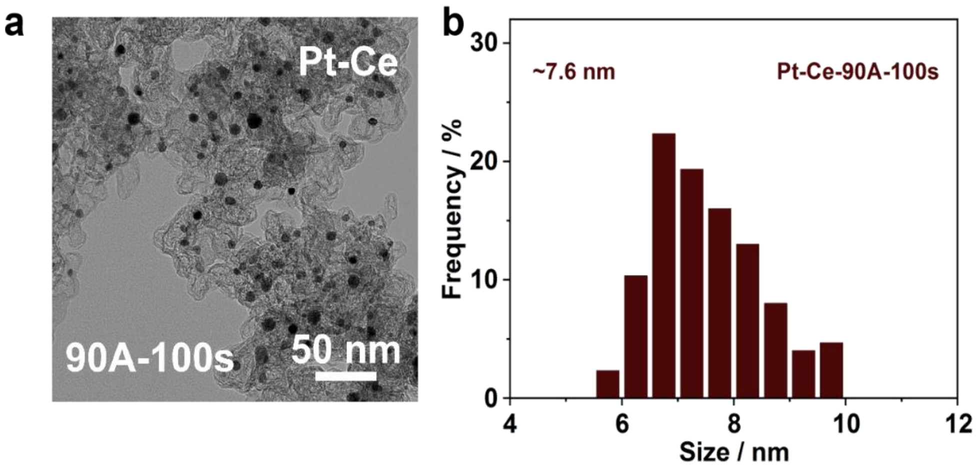

Figure S10. (a) The TEM image of Pt-Ce-90A-100s. (b) The histogram of particle size distribution of Pt-Ce-90A-100s.

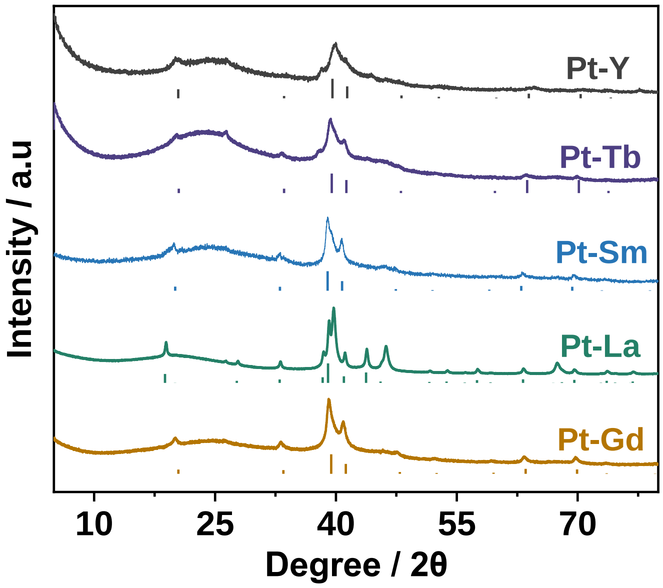

Figure S11. The XRD patterns of five Pt-RE alloys. The reference patterns are Pt_2Y (JCPDS 65-8050), Pt_2Tb (JCPDS 19-0912), Pt_2Sm (JCPDS 65-8177), Pt_5La (JCPDS 65-9345), Pt_2Gd (JCPDS 65-1745).

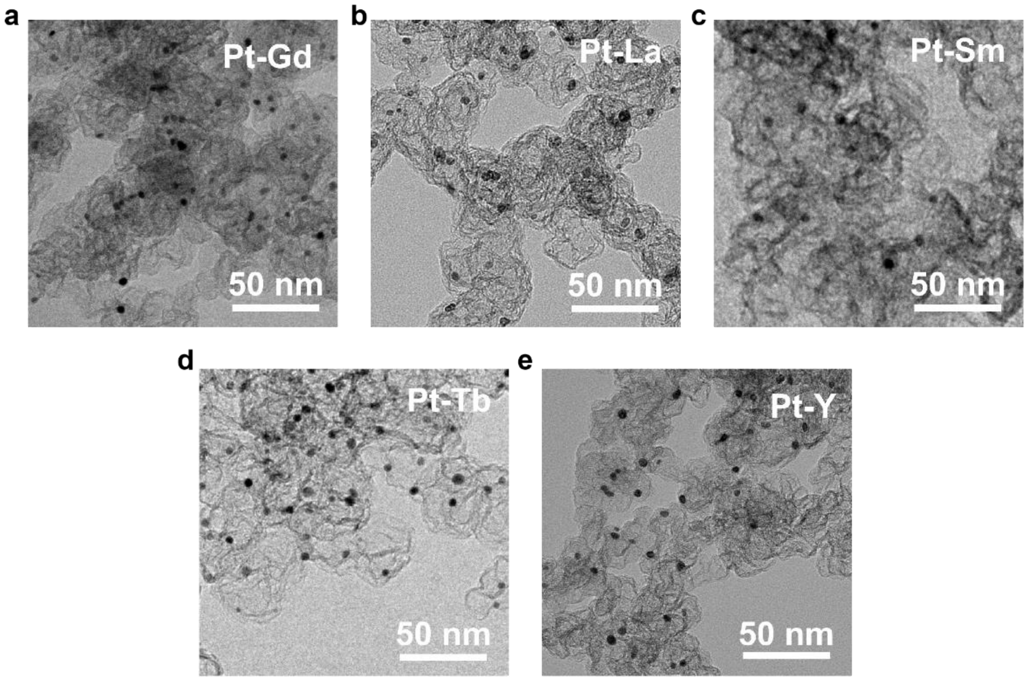

Figure S12. The TEM images of (a) Pt-Gd-80A-100s (b) Pt-La-80A-100s (c) Pt-Sm-80A-100s (d) Pt-Tb-80A-100s (e) Pt-Y-80A-100s.

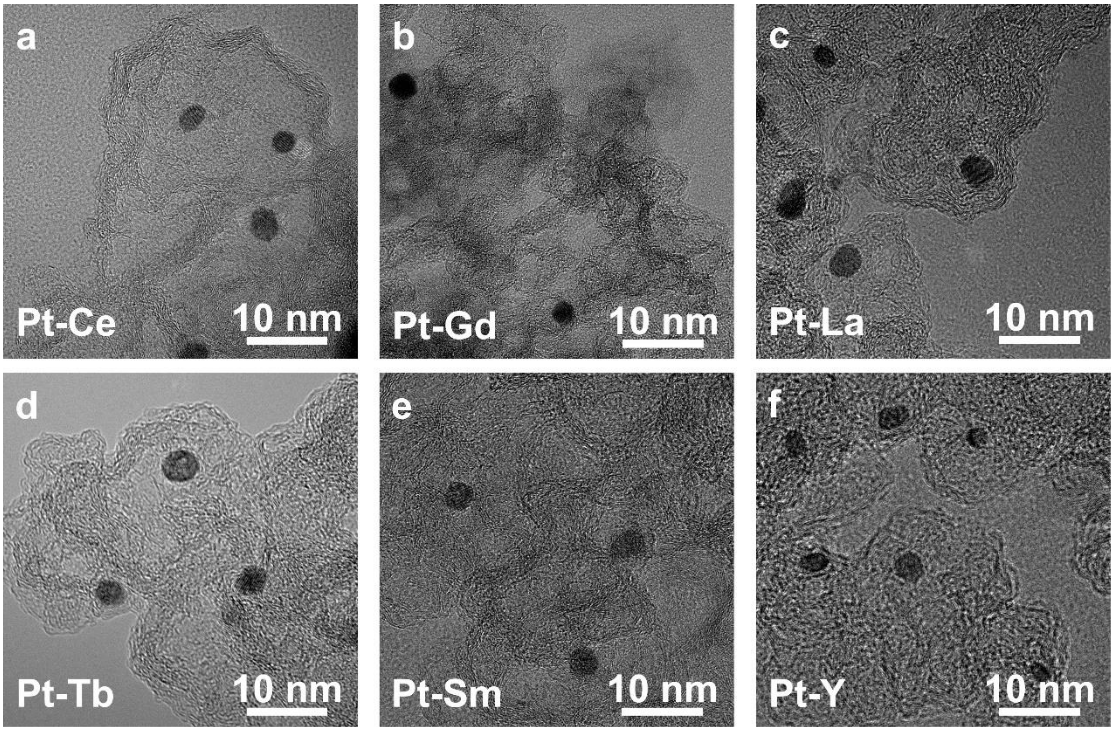

Figure S13. HRTEM images of (a) Pt-Ce (b) Pt-Gd (c) Pt-La (d) Pt-Tb (e) Pt-Sm (f) Pt-Y synthesize at 80 A for 100 s.

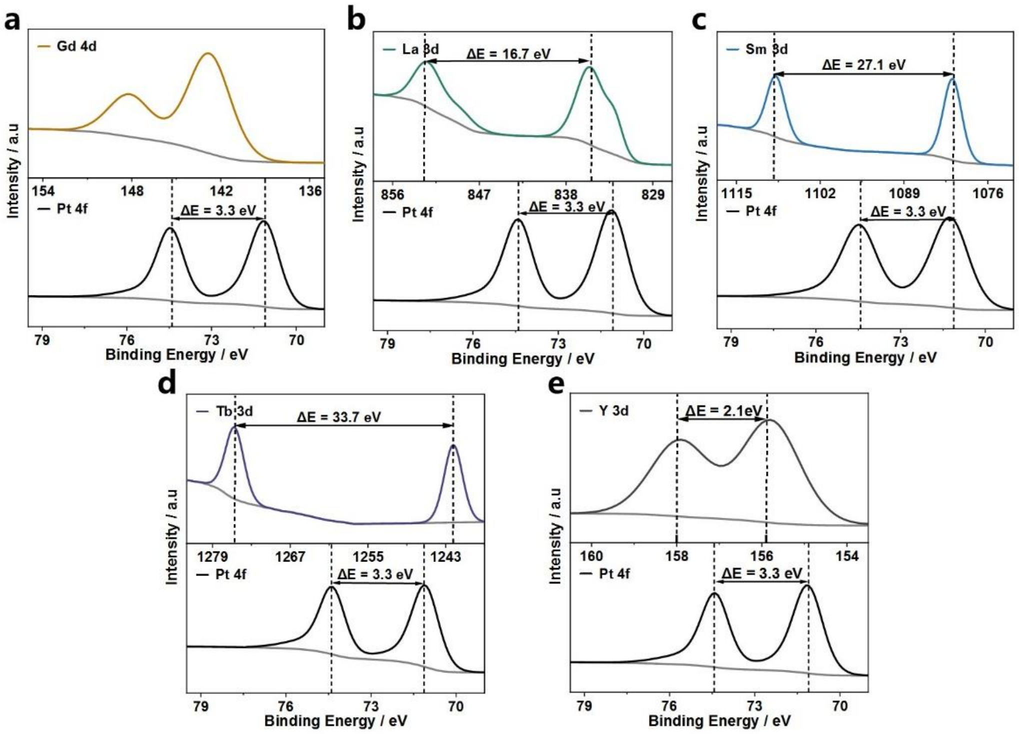

Figure S14. (a) The XPS spectra of Gd 4d and Pt 4f. (b) The XPS spectra of La 3d and Pt 4f. (c) The XPS spectra of Sm 3d and Pt 4f. (d) The XPS spectra of Tb 3d and Pt 4f. (e) The XPS spectra of Y 3d and Pt 4f.

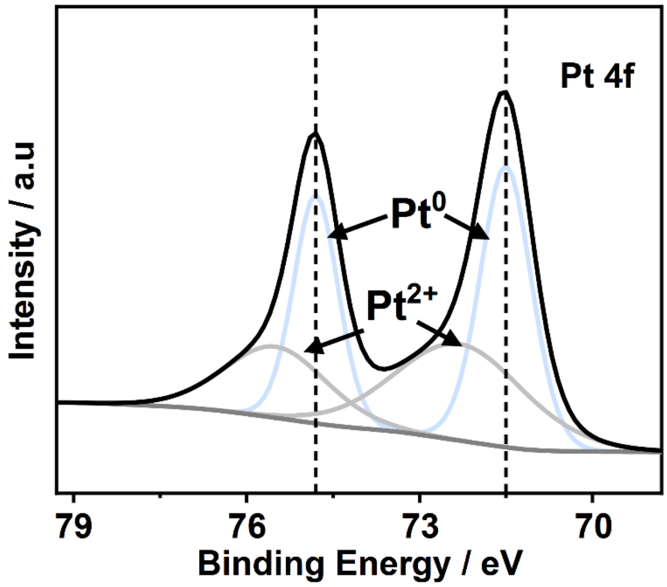

Figure S15. The XPS Pt 4f spectrum of JM Pt/C.

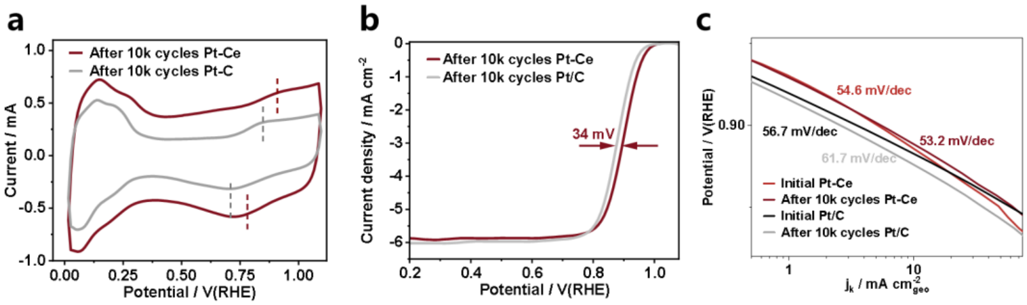

Figure S16. (a) CV curves of Pt-Ce and Pt/C after 10k cycles obtained in N_2-saturated 0.1 M $HClO_4$. (b) LSV curves of Pt-Ce and Pt/C after 10k cycles obtained at 1600 rpm in 0.1 M $HClO_4$. (c) The Tafel plots of Pt-Ce and Pt/C obtained at 0.9 V before and after ADT test.

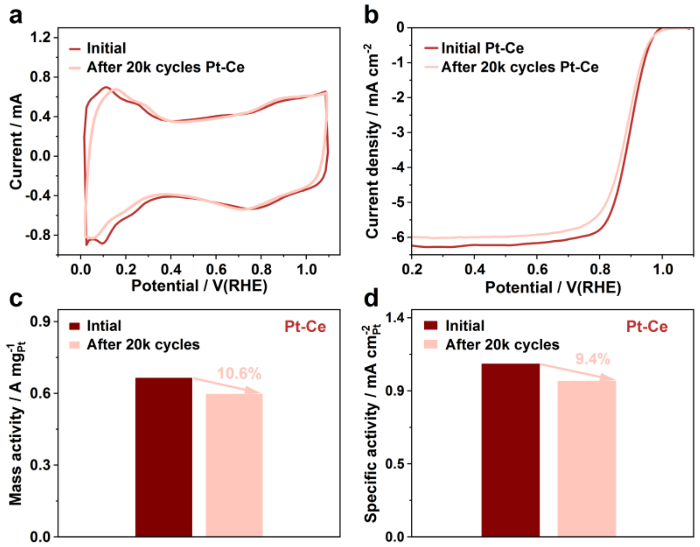

Figure S17. (a) CV curves of Pt-Ce after 20k cycles in N_2-saturated 0.1 M $HClO_4$. (b) LSV curves of Pt-Ce after 20k cycles obtained at 1600 rpm in 0.1 M $HClO_4$. (c) Mass activity and (d) specific activity values obtained at 0.9 V before and after ADT test.

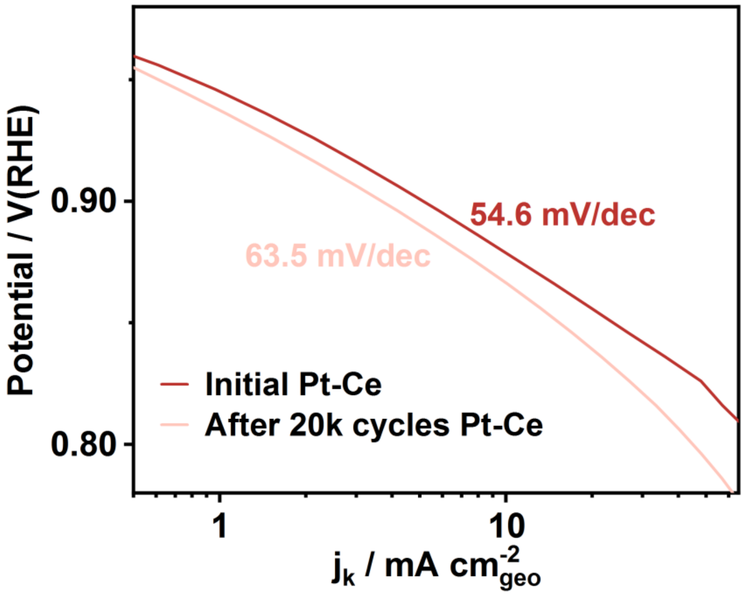

Figure S18. The Tafel plots of Pt-Ce obtained at 0.9 V before and after ADT test.

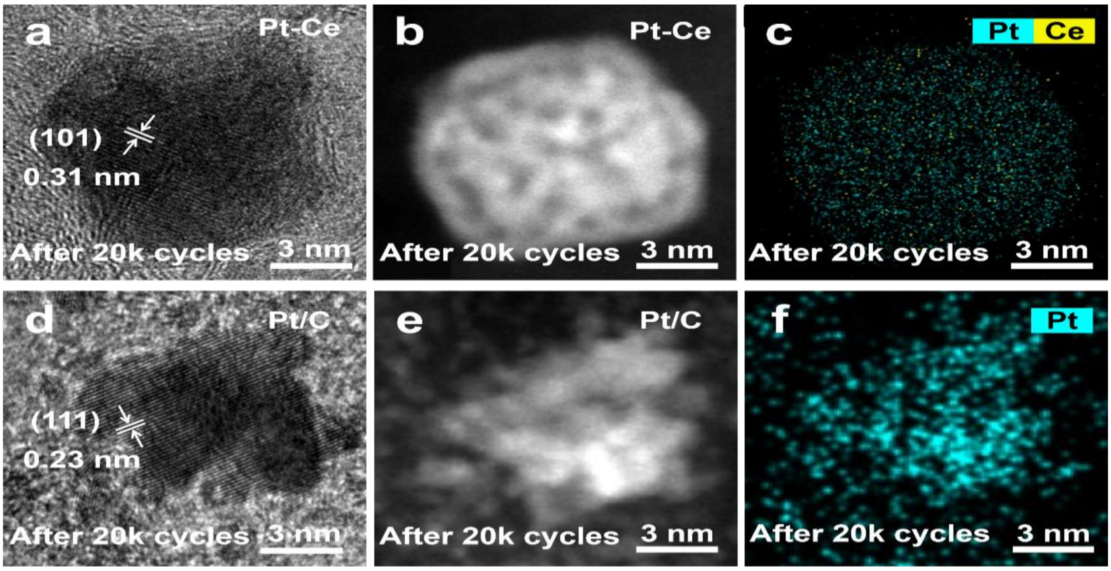

Figure S19. (a) The HR-TEM image of Pt-Ce-80A-100s after 20k cycles. (b) The HAADF-STEM image of Pt-Ce-80A-100s after 20k cycles. (c) The HAADF-STEM elements mapping. (d) The HR-TEM image of Pt/C after 20k cycles of ADT test. (e) The HAADF-STEM image of Pt/C after 20k cycles. (f) HAADF-STEM elements mapping.

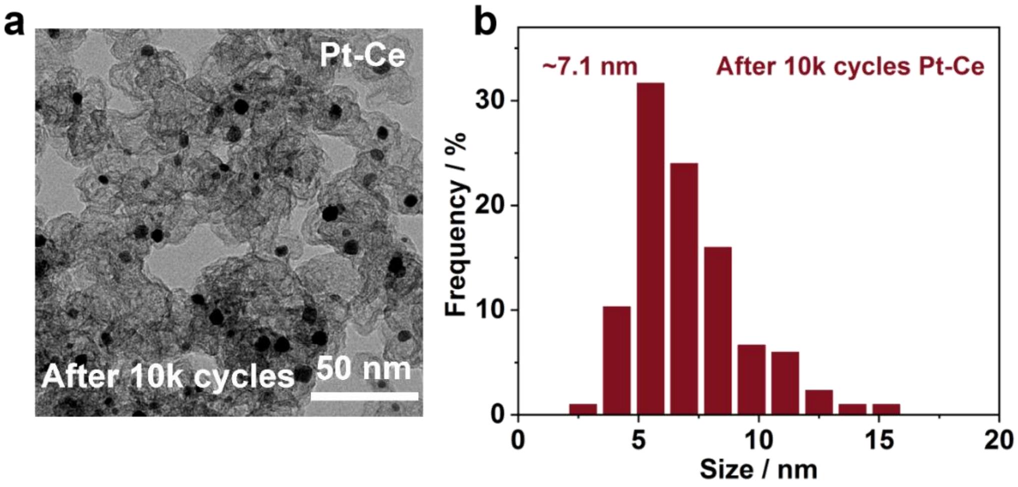

Figure S20. The TEM image of Pt-Ce-80A-100s after 10k ADT cycles test.

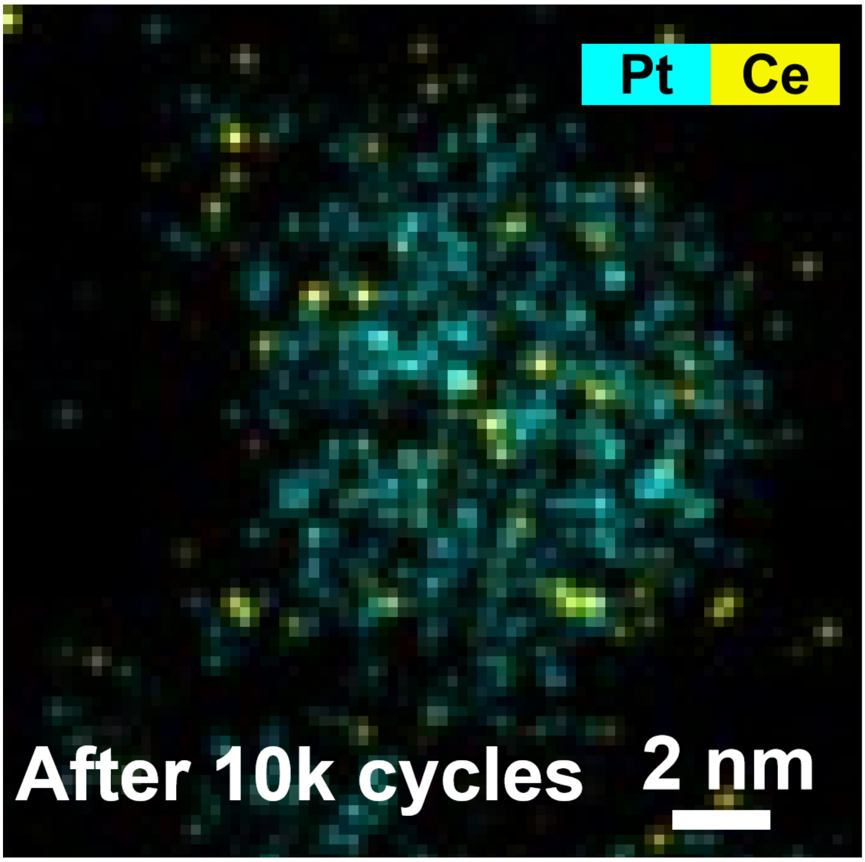

Figure S21. The HAADF-STEM elements mapping of Pt-Ce-80A-100s after 10k ADT cycles test.

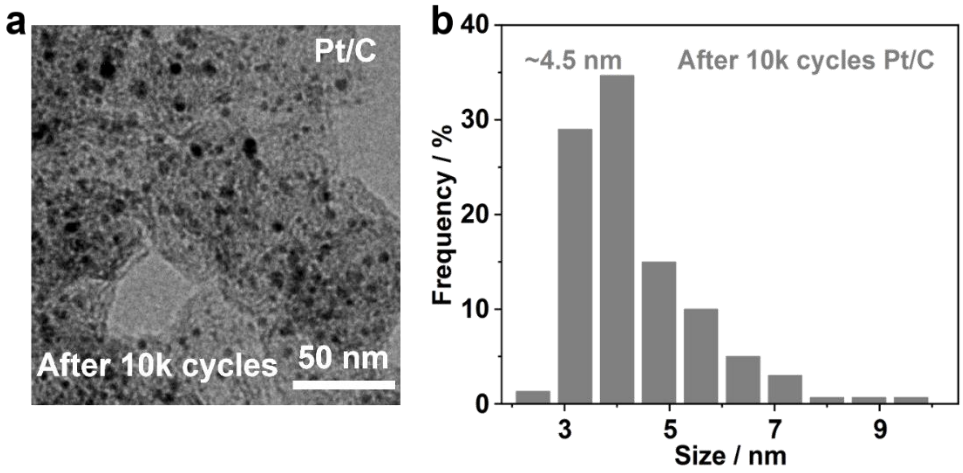

Figure S22. The TEM image of Pt/Ce after 10k ADT cycles test.

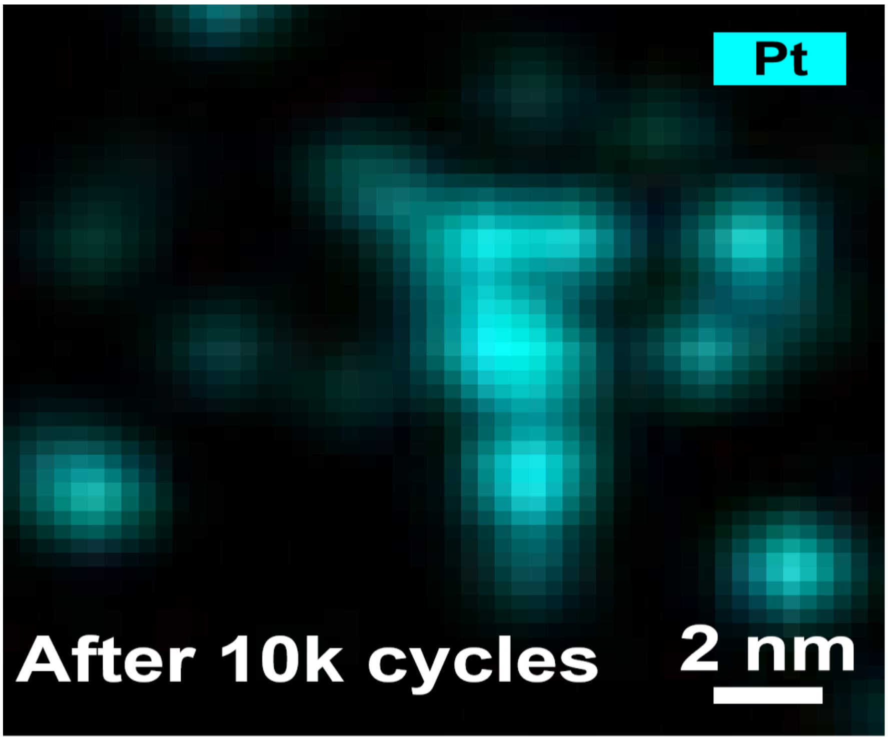

Figure S23. The HAADF-STEM elements mapping of Pt/C after 10k ADT cycles test.

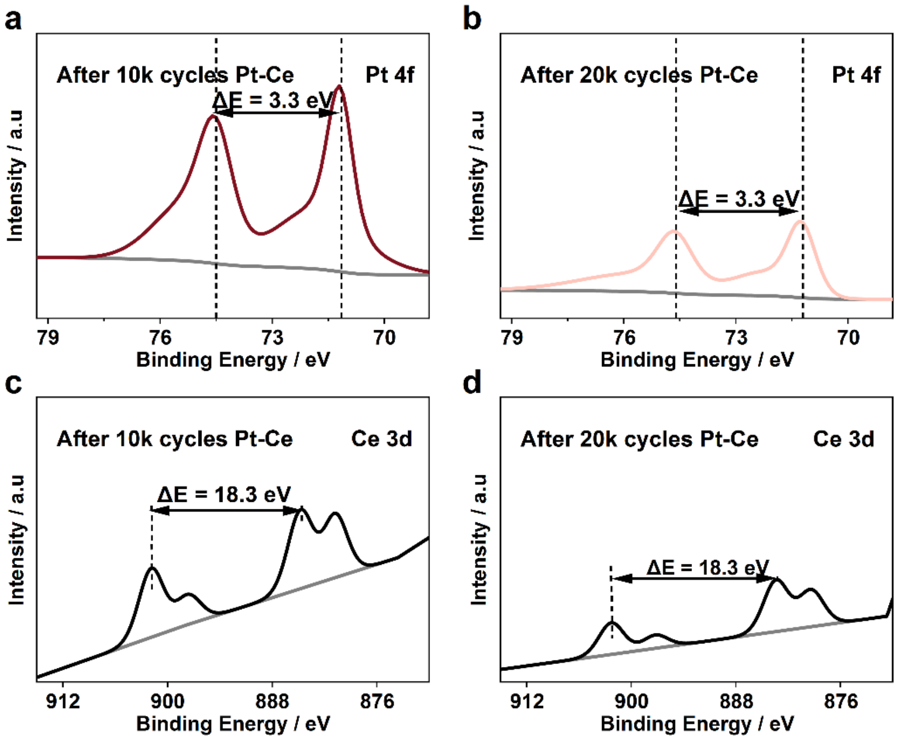

Figure S24. (a) The XPS Pt 4f spectrum of Pt-Ce after 10k cycles. (b) The XPS Pt 4f spectrum of Pt-Ce after 20k cycles. (c) The XPS Ce 3d spectrum of Pt-Ce after 10k cycles. (d) The XPS Ce 3d spectrum of Pt-Ce after 20k cycles.

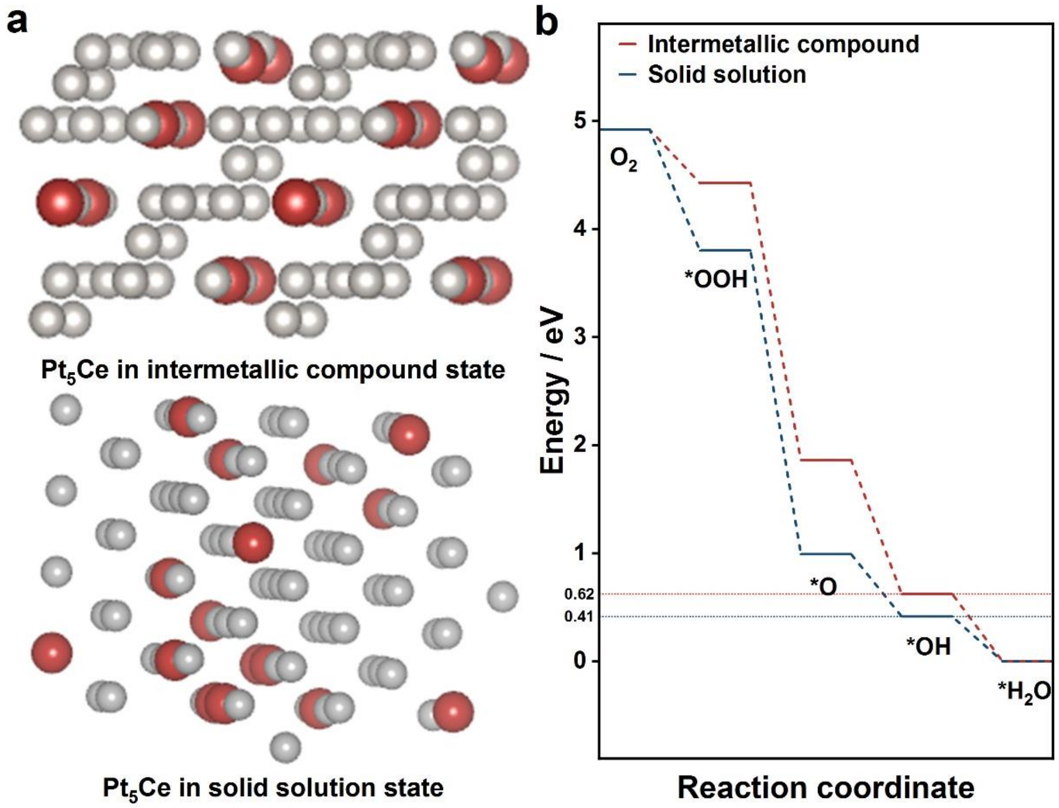

Figure S25. (a) Schematic illustration of Pt_5Ce in the states of intermetallic compound and solid solution. (b) Free energy diagrams for the four-electron associative pathway of ORR on Pt (111) in the intermetallic compound and solid solution at potential with respect to the reversible hydrogen electrode: 0 V.

In Figure S25a, we constructed two Pt-Ce models that includes both intermetallic compound and solid solution. In Figure S25b, at potential of U=0 V, the potential value of the rate-determining step in intermetallic Pt_5Ce compound is about 0.62 eV, which is larger than that in solid solution (0.41 eV), indicating the lower adsorption energies of ORR intermediates. This result reveals that the ordered coordination effect of Ce on Pt and the electron transfer from Ce to Pt generated by intermetallic compounds are beneficial for improving ORR performance. Therefore, the ORR performance of Pt_5Ce alloys in intermetallic compound state is better than that in solid solution state.

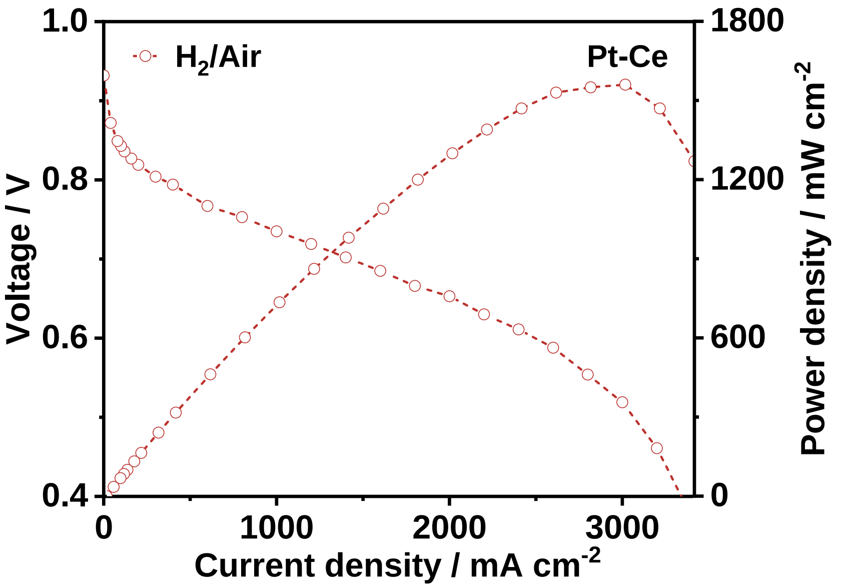

Figure S26. The curves of i−V polarizations and power density of MEA operated in H_2/Air. The results show that the fuel cell utilizing Pt-Ce alloy at the cathode exhibits a current density of 2.12 A/cm^2 at 0.65 V and a peak power density of 1.62 W/cm^2. The above power density accounts for nearly 1.4 times than the commercial Pt/C-based MEA.

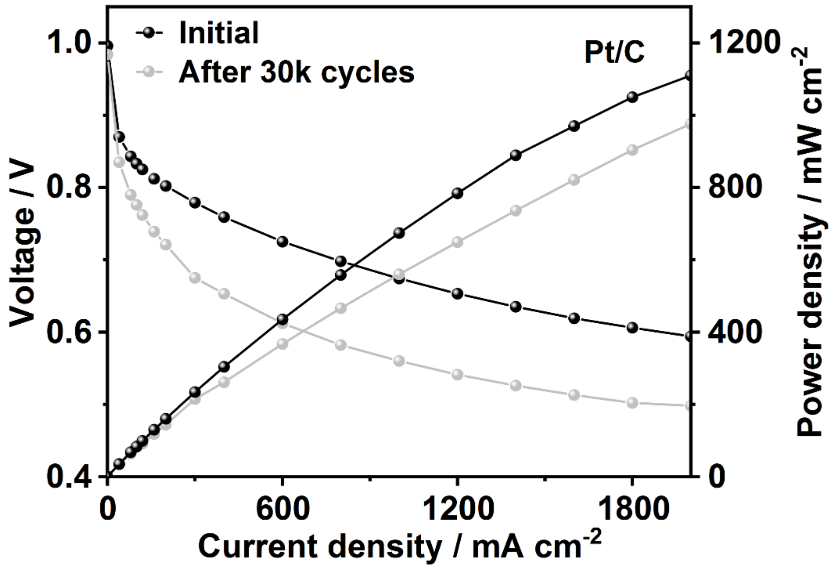

Figure S27. H_2-O_2 fuel cell i−V polarization and power density curves of MEA initial and after 30k cycles with Pt loading of 0.1 mg_{Pt} cm^{-2} for Pt/C at the cathode and anode.

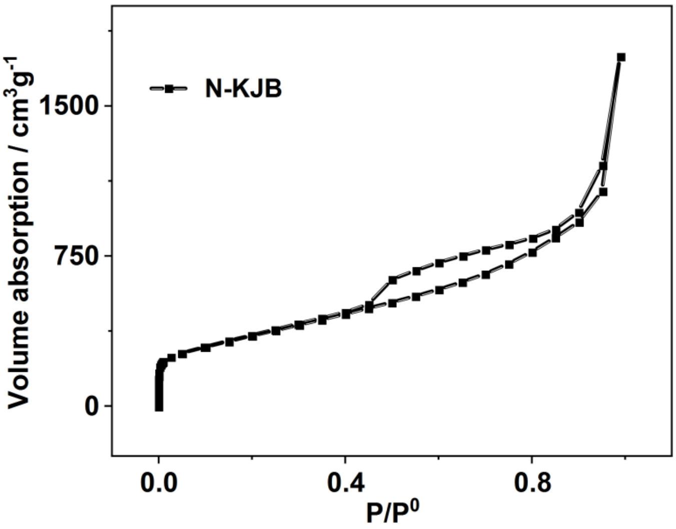

Figure S28. The N_2 absorption-desorption isothermals of the carbon support N-KJB.

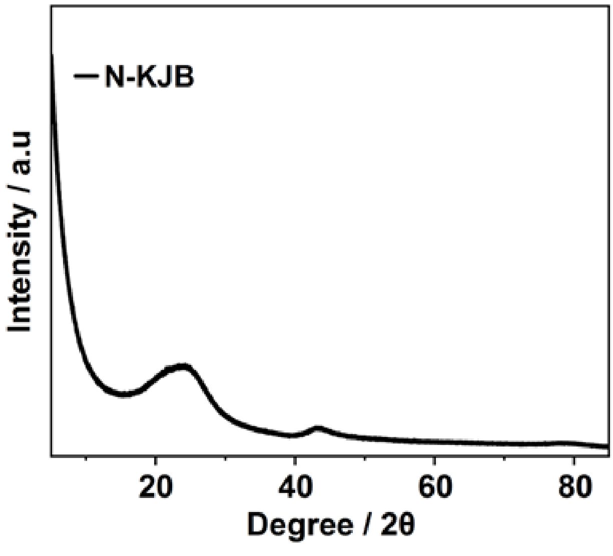

Figure S29. The XRD pattern of N-KJB.

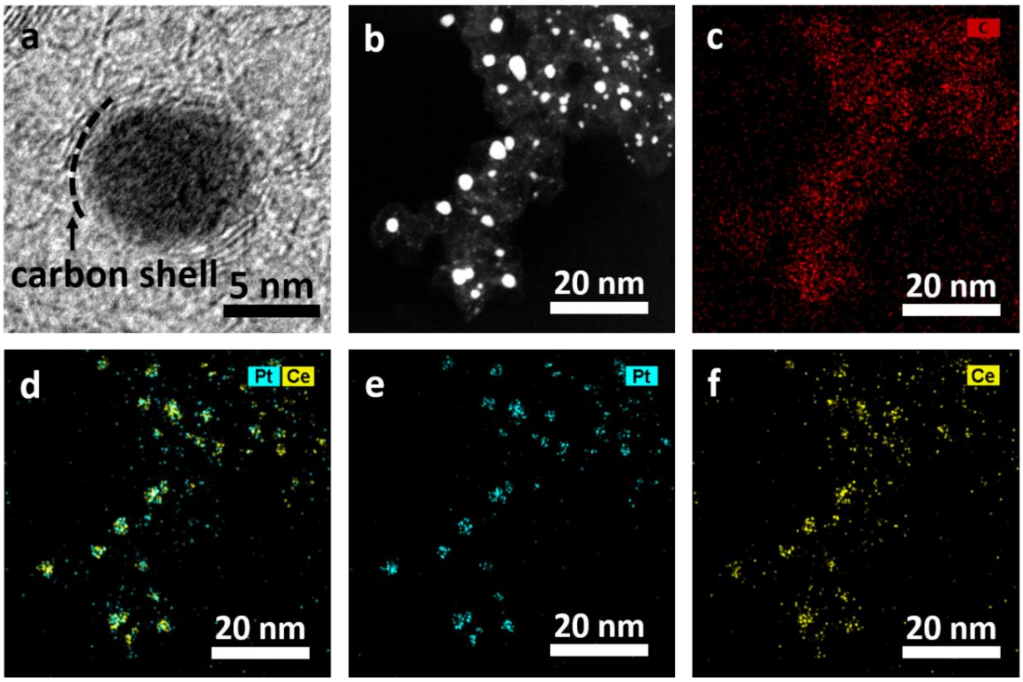

Figure S30. (a) The TEM image of Pt-Ce with carbon shell wrapping. (b) The HAADF-STEM image of Pt-Ce with carbon shell wrapping. (c-f) HAADF-STEM elements mapping.

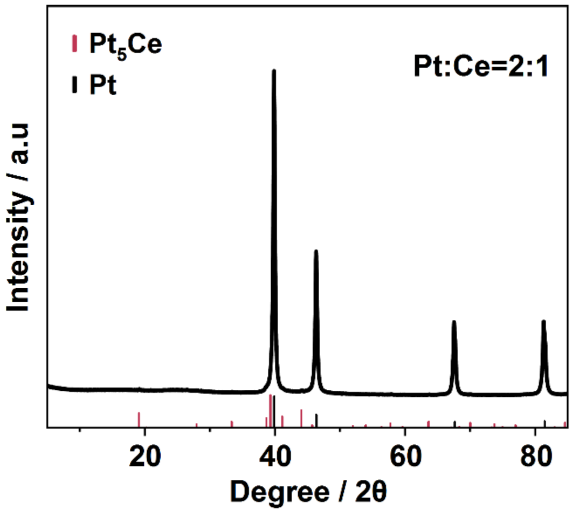

Figure S31. The XRD pattern of Pt-Ce, which atom ratio off is 2:1.

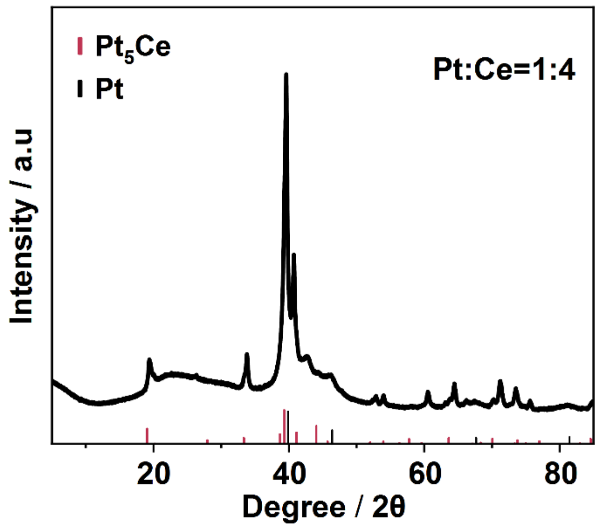

Figure S32. The XRD pattern of Pt-Ce, which atom ratio off is 1:4.

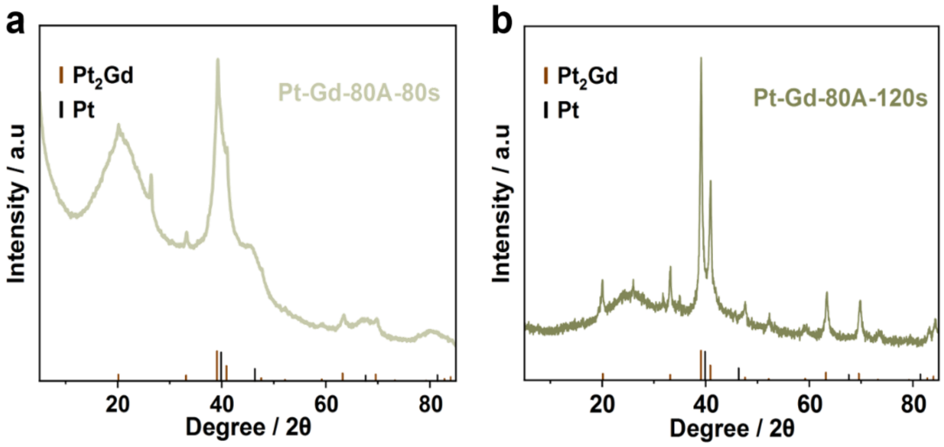

Figure S33. The XRD pattern of Pt-Gd-80A-80s.

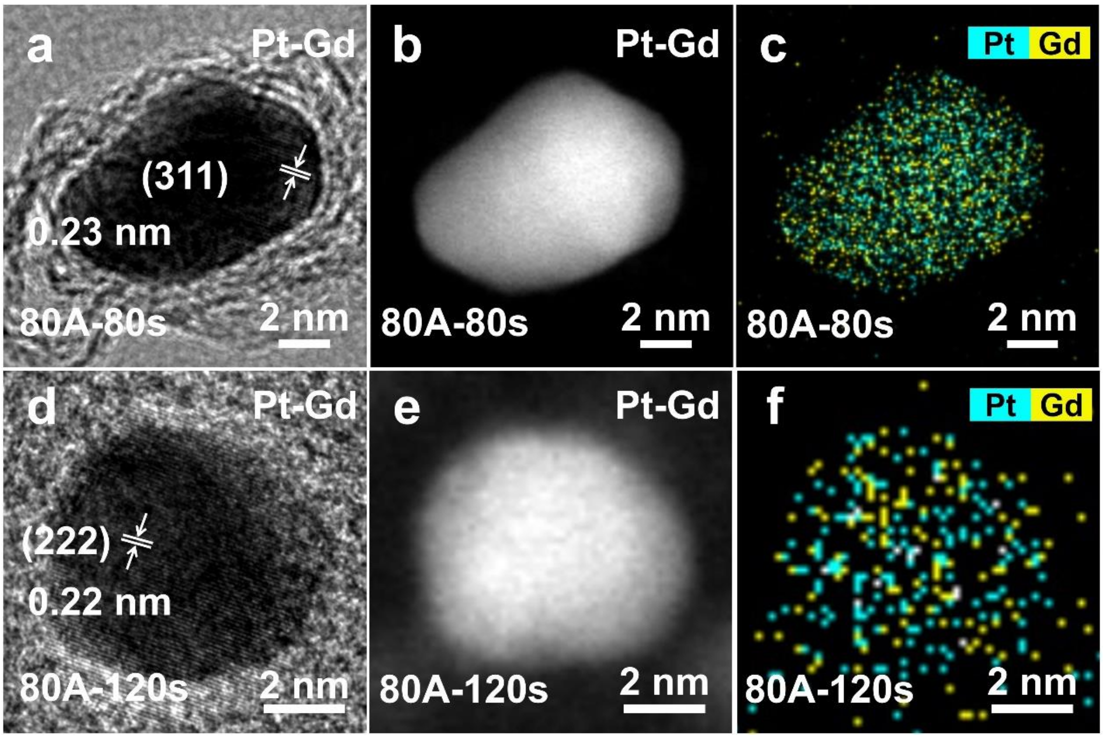

Figure S34. (a) The HR-TEM image of Pt-Gd-80A-80s. (b) The HAADF-STEM image of Pt-Gd-80A-80s. (c) The HAADF-STEM elements mapping Pt-Gd-80A-80s. (d) The HR-TEM image of Pt-Gd-80A-120s. (e) The HAADF-STEM image of Pt-Gd-80A-120s. (f) The HAADF-STEM elements mapping Pt-Gd-80A-120s.

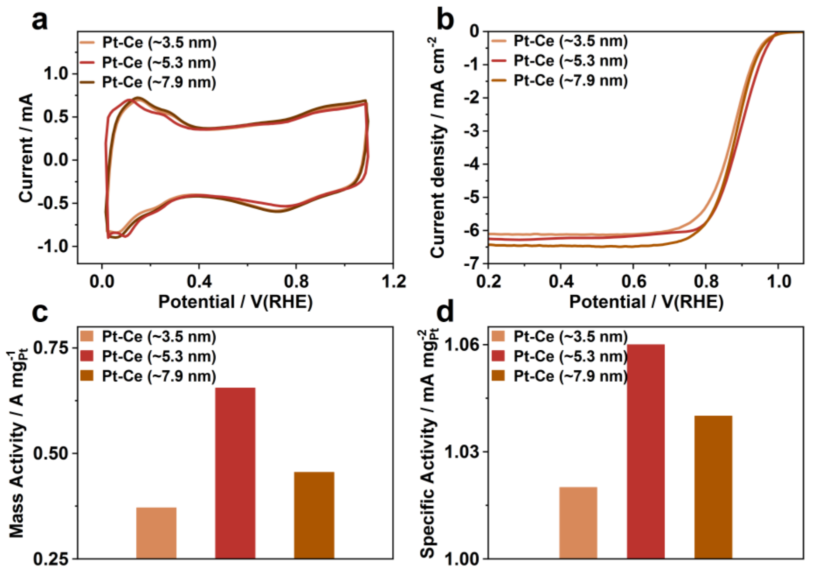

Figure S35. (a) CV curves of Pt-Ce which in different particle size obtained in N_2-saturated 0.1 M $HClO_4$. (b) LSV curves of Pt-Ce at 1600 rpm in 0.1 M $HClO_4$. (c) Mass activity and (d) specific activity values obtained at 0.9 V. The peak of Pt-Ce (~5.3 nm) compared with the other two in highest potential, indicating that the alloying with Ce resulted in a greater extent down shift of d-band center of Pt, thus the bonding strength between Pt and oxygenated intermediates was weakened, promoting the ORR progress and improving the activity.

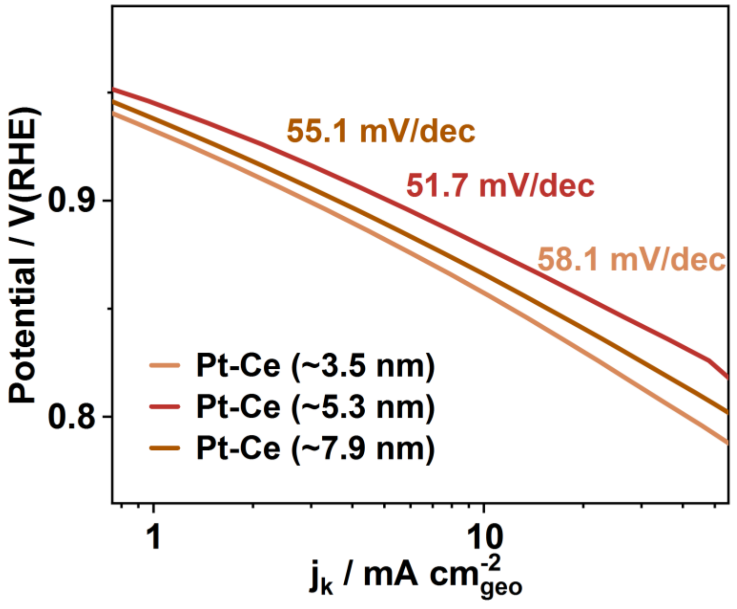

Figure S36. The Tafel plots of Pt-Ce which in different particle size.

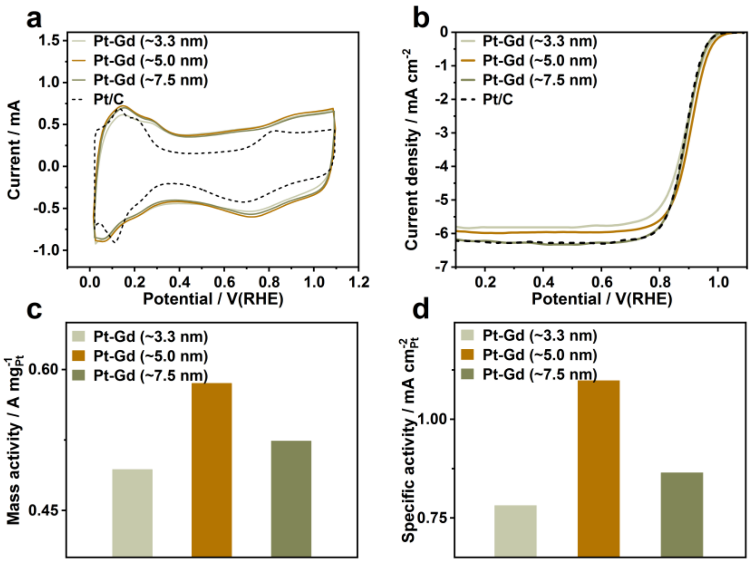

Figure S37. (a) CV curves of Pt-Gd which in different particle size obtained in N_2-saturated 0.1 M $HClO_4$. (b) LSV curves of Pt-Gd at 1600 rpm in 0.1 M $HClO_4$. (c) Mass activity and (d) specific activity values obtained at 0.9 V.

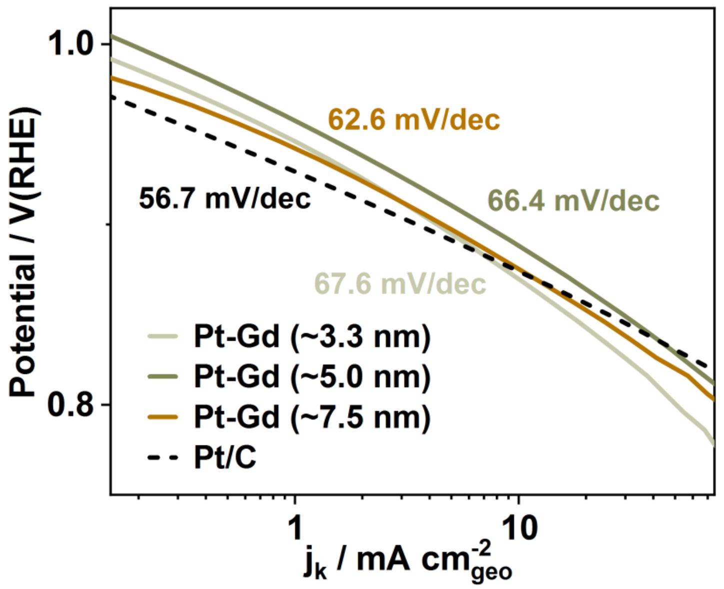

Figure S38. The Tafel plots of Pt-Gd which in different particle size.

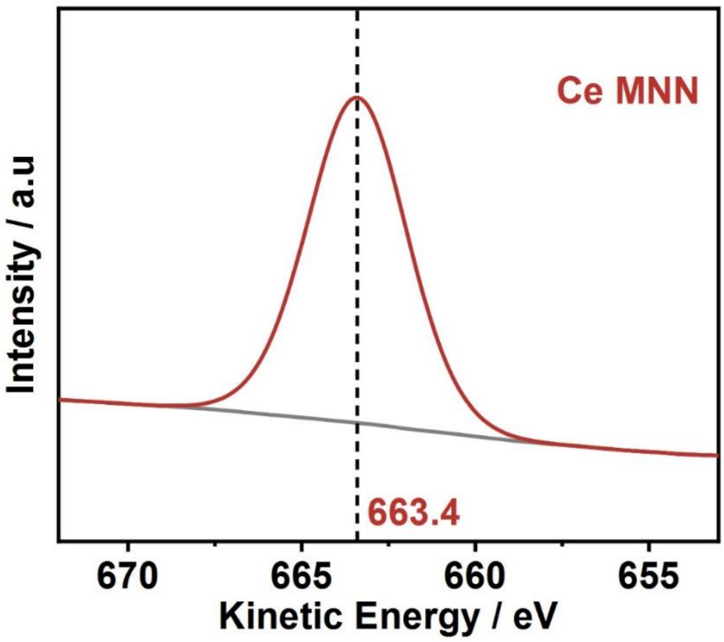

Figure S39. The auger spectrum of Ce MNN, further confirming the metallic state of Ce.

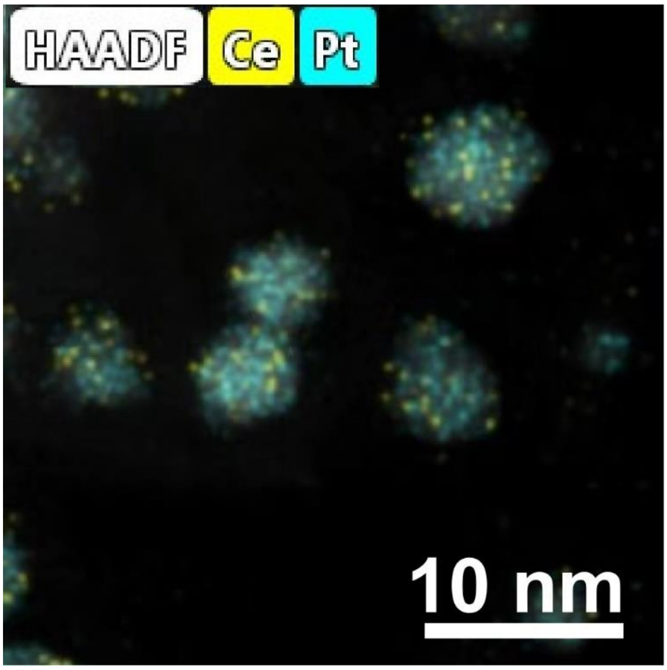

Figure S40. The HAADF-STEM elements mapping at low magnification. It is clear from this image that Pt and Ce are well-dispersed throughout the alloy.

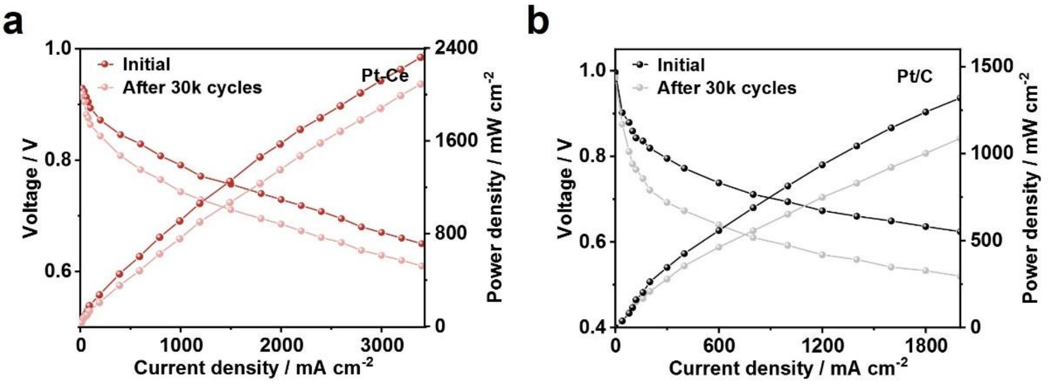

Figure S41. I−V polarization and power density curves of H_2-O_2 fuel cell utilizing Nafion 211 (a) Pt-Ce. (b) Pt/C.

The fuel cell utilizing Pt-Ce alloy at the cathode exhibits a current density of 3.41 A/cm^2 at 0.65 V and a peak power density of 2.32 W/cm^2. The MA of Pt-Ce in the PEMFC is 0.92 A/mg$_{Pt}$ at 0.9 V, with 2.09 times higher than that of the DOE 2025 activity target. After the stability test of 30k cycles, the peak power density shows a decrease of 10.4% to 2.08 W/cm^2. And MA decreased 12.1% to 0.81 A/mg$_{Pt}$ after ADT.

The fuel cell utilizing Pt/C alloy at the cathode exhibits a current density of 1.6 A/cm^2 at 0.65 V and a peak power density of 1.32 W/cm^2. Its MA values 0.42 A/mg$_{Pt}$ at 0.9 V. After the stability test of 30k cycles, the peak power density decreases 18% to 1.08 W/cm^2, and MA decreases 38.1% to 0.26 A/mg$_{Pt}$.

Supplementary references

[1] D.D. Sarma, C. Carbone, R. Cimino, P, Sen, W. Gudat, W. Eberhardt, P*hys. Rev. B.* **1993**,47,4853.

[2] D. Strmcnik, M. Escudero-Escribano, K. Kodama, V. R. Stamenkovic, A. Cuesta and N. M. Marković, *Nat. Chem.* **2010**, 2, 880.

[3] H. A. Hansen, V. Viswanathan and J. K. Nørskov, *J. Phys. Chem. C*, **2014**, 118, 6706.

[4] D. Macciò, F. Rosalbino, A. Saccone, S. Delfino, *J. Alloys Compd.* **2005**, 391, 60.

[5] T. P. Johansson, E. T. Ulrikkeholm, P. Hernandez-Fernandez, P. Malacrida, H. A. Hansen, A. S. Bandarenka, J. K. Nørskov, J. Rossmeisl, I. E. L. Stephens, I. Chorkendorff, *Top. Catal.* **2013**, 57, 245.

[6] S. J. Yoo, S. J. Hwang, J.-G. Lee, S.-C. Lee, T.-H. Lim, Y.-E. Sung, A. Wieckowski, S.-K. Kim, *Energy Environ. Sci.* **2012**, 5,7521.
[7] A. Velázquez-Palenzuela, F. Masini, A. F. Pedersen, M. Escudero-Escribano, D. Deiana, P. Malacrida, T. W. Hansen, D. Friebel, A. Nilsson, I. E. L. Stephens, I. Chorkendorff, *J. Catal.* **2015**, 328, 297.
[8] J. Fichtner, B. Garlyyev, S. Watzele, H. A. El-Sayed, J. N. Schwammlein, W. J. Li, F. M. Maillard, L. Dubau, J. Michalicka, J. M. Macak, A. Holleitner, A. S. Bandarenka, *ACS Appl. Mater. Interfaces* **2019**, 11, 5129.
[9] C. Roy, B. P. Knudsen, C. M. Pedersen, A. Velázquez-Palenzuela, L. H. Christensen, C. D. Damsgaard, I. E. L. Stephens, I. Chorkendorff, *ACS Catal.* **2018**, 8, 2071.
[10] Y. Hu, J. O. Jensen, L. N. Cleemann, B. A. Brandes, Q. Li, *J. Am. Chem. Soc.* **2020**, 142, 953.
[11] Xu. S.-L, Zhao. S, Zeng. W.-J, Li. S, Zuo, M. Lin. Y, Chu, S, Chen, P. Liu. J, Liang. H.-W. *Chem. Mate*r. **2022**, 34, 10789.
[12] Campos-Roldán. C. A, Parnière. A, Donzel. N, Pailloux. F, Blanchard. P.-Y, Jones. D. J, Rozière. J, Cavaliere. S. *ACS Appl. Energy Mater.* **2022**, 5, 3319.
[13] Jiang. Y, Fu. T, Liu. J, Zhao. J, Li. B, Chen. Z. *RSC Adv.* **2022**, 12, 4805.
[14] Zhang. S, Zeng. Z, Li. Q, Huang. B, Zhang. X, Du. Y, Yan. C.-H. *Energy Environ. Sci.* **2021**, 14, 5911.
[15] K. Ichihashi, S. Muratsugu, H. Matsui, K. Higashi, O. Sekizawa, T. Uruga, M. Tada, *J. Phys. Chem. C.* **2020**, 124, 26925.
[16] M. Escudero-Escribano, A. Verdaguer-Casadevall, P. Malacrida, U. Gronbjerg, B. P. Knudsen, A. K. Jepsen, J. Rossmeisl, I. E. Stephens, I. Chorkendorff, *J. Am. Chem. Soc.* **2012**, 134, 16476.
[17] P. Malacrida, M. Escudero-Escribano, A. Verdaguer-Casadevall, I. E. L. Stephens, I. Chorkendorff, *J. Mater. Chem. A* **2014**, 2,4234.
[18] M. Escudero-Escribano, P. Malacrida, M. H. Hansen, U. G. Vej-Hansen, A. Velazquez-Palenzuela, V. Tripkovic, J. Schiotz, J. Rossmeisl, I. E. Stephens, I. Chorkendorff, *Science* **2016**, 352, 73.
[19] Y. Luo, K. Li, Y. Chen, J. Feng, L. Wang, Y. Jiang, L. Li, G. Yu, J. Feng, *Adv Mater* **2023**, e2300624.
[20] G. Feng, F. Ning, Y. Pan, T. Chen, J. Song, Y. Wang, R. Zou, D. Su, D. Xia, *J. Am. Chem. Soc.* **2023**.
[21] F. Xiao, Q. Wang, G.-L. Xu, X. Qin, I. Hwang, C.-J. Sun, M. Liu, W. Hua, H.-w. Wu, S. Zhu, J.-C. Li, J.-G. Wang, Y. Zhu, D. Wu, Z. Wei, M. Gu, K. Amine, M. Shao, *Nat. Catal.* **2022**, 5, 503.
[22] Q. Q. Cheng, S. Yang, C. H. Fu, L. L. Zou, Z. Q. Zou, Z. Jiang, J. L. Zhang, H. Yang, *Energy Environ. Sci.* **2022**, 15, 278.
[23] M. Zhu, C. Zhao, X. Liu, X. Wang, F. Zhou, J. Wang, Y. Hu, Y. Zhao, T. Yao, L.-M. Yang, Y. Wu, *ACS Catal.* **2021**, 11, 3923.
[24] C. L. Yang, L. N. Wang, P. Yin, J. Liu, M. X. Chen, Q. Q. Yan, Z. S. Wang, S. L. Xu, S. Q. Chu, C. Cui, H. Ju, J. Zhu, Y. Lin, J. Shui, H. W. Liang, *Science* **2021**, 374, 459.

[25] X. Duan, F. Cao, R. Ding, X. Li, Q. Li, R. Aisha, S. Zhang, K. Hua, Z. Rui, Y. Wu, J. Li, A. Li, J. Liu, *Adv. Energy Mater.* **2022**, 12,2103144.
[26] C. Zhan, H. Sun, L. Lü, L. Bu, L. Li, Y. Liu, T. Yang, W. Liu, X. Huang, *Adv. Funct. Mater.* **2022**, 33,2212442.
[27] Z. Chen, C. Hao, B. Yan, Q. Chen, H. Feng, X. Mao, J. Cen, Z. Q. Tian, P. Tsiakaras, P. K. Shen, *Adv. Energy Mater.* **2022**, 12,2201600.
[28] J. Lin, J. Wang, Y. Wu, P. Yang, Q. Liu, M. Li, S. Du, R. Chen, L. Tao, *Chem. Asian J.* **2023**, 18, e202300137.
